# Protein–Ligand Binding Free-Energy Calculations with ARROW—A Purely First-Principles Parameterized Polarizable Force Field

**DOI:** 10.1021/acs.jctc.2c00930

**Published:** 2022-12-02

**Authors:** Grzegorz Nawrocki, Igor Leontyev, Serzhan Sakipov, Mikhail Darkhovskiy, Igor Kurnikov, Leonid Pereyaslavets, Ganesh Kamath, Ekaterina Voronina, Oleg Butin, Alexey Illarionov, Michael Olevanov, Alexander Kostikov, Ilya Ivahnenko, Dhilon S. Patel, Subramanian K. R. S. Sankaranarayanan, Maria G. Kurnikova, Christopher Lock, Gavin E. Crooks, Michael Levitt, Roger D. Kornberg, Boris Fain

**Affiliations:** † InterX Inc., 805 Allston Way, Berkeley California, 94710, United States; ‡ Faculty of Physics, Lomonosov Moscow State University, Moscow 119991, Russia; § Department of Chemistry, Carnegie Mellon University, Pittsburgh, Pennsylvania 15213, United States; ∥ Center for Nanoscale Materials, Argonne National Lab, Lemont, Illinois 60439, United States; ⊥ Department of Mechanical and Industrial Engineering, University of Illinois, Chicago, Illinois 60607, United States; # Department of Neurology and Neurological Sciences, Stanford University School of Medicine, Palo Alto, California 94304, United States; ∇ Department of Structural Biology, Stanford University School of Medicine, Stanford, California 94305, United States

## Abstract

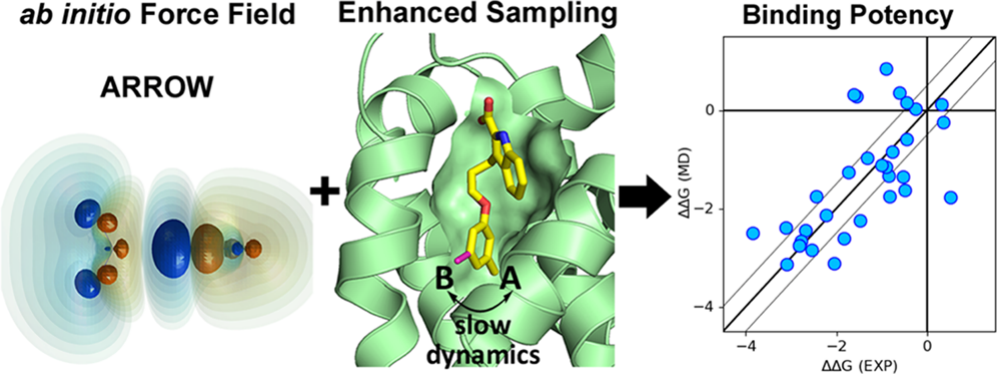

Protein–ligand binding free-energy calculations using molecular dynamics (MD) simulations have emerged as a powerful tool for in silico drug design. Here, we present results obtained with the ARROW force field (FF)—a multipolar polarizable and physics-based model with all parameters fitted entirely to high-level ab initio quantum mechanical (QM) calculations. ARROW has already proven its ability to determine solvation free energy of arbitrary neutral compounds with unprecedented accuracy. The ARROW FF parameterization is now extended to include coverage of all amino acids including charged groups, allowing molecular simulations of a series of protein–ligand systems and prediction of their relative binding free energies. We ensure adequate sampling by applying a novel technique that is based on coupling the Hamiltonian Replica exchange (HREX) with a conformation reservoir generated via potential softening and nonequilibrium MD. ARROW provides predictions with near chemical accuracy (mean absolute error of ∼0.5 kcal/mol) for two of the three protein systems studied here (MCL1 and Thrombin). The third protein system (CDK2) reveals the difficulty in accurately describing dimer interaction energies involving polar and charged species. Overall, for all of the three protein systems studied here, ARROW FF predicts relative binding free energies of ligands with a similar accuracy level as leading nonpolarizable force fields.

## Introduction

Free-energy calculations of ligand binding to a protein can serve as a powerful tool for structure-based small-molecule drug design, especially at the stages of lead selection (hit-to-lead) and lead optimization, where ligands of high binding affinity are desired. A change of free energy, Δ*G*, upon binding quantitatively describes ligand–protein affinity. In silico calculations of protein–ligand binding Δ*G* have numerous advantages over expensive experimental approaches. The calculations can be performed in a fully automated manner; consequently, a large number of ligands can be evaluated and numerous drug candidates with diverse structures can be selected. The relative energy, ΔΔ*G*, i.e., calculation of Δ*G* of one ligand with respect to another, is usually sufficient to guide the process of ligand optimization and can be calculated more accurately than the absolute energy via alchemical transformations.^1,2^

In recent years, calculations of binding free energies of ligands in proteins, using all-atom force fields, have grown in importance. Effective software packages have been developed to perform such calculations.^3−5^ All-atom force fields used in these calculations^6−8^ typically combine parameters derived from quantum mechanics (QM) calculations and empirical parameters fitted to reproduce certain experimental observables. However, systematic studies performed on a large set of protein–ligand systems suggest that current methodologies may have reached a limit of about 1 kcal/mol mean absolute error (MAE) from the experiment.^9−12^ There are two factors responsible for the low accuracy, insufficient quality of molecular force fields (FF), and poor conformational sampling.

Most of the currently available force fields are all-atom fixed-charge models, e.g., AMBER/GAFF,^6^ CHARMM/CGenFF,^13^ OPLS,^10,14,15^ GROMOS,^16^ and MMFF.^17^ Although they are well established, highly refined, and computationally efficient, it is generally accepted that they are not sophisticated enough to describe complicated protein–ligand interactions with an accuracy required for drug design. Specifically, they do not allow an accurate description of electrostatic and exchange interactions in the proteins, or nonadditive effects of atomic interactions, e.g., electronic polarizability. The absence of electronic polarizability significantly limits the force field’s ability to correctly describe highly heterogeneous environments such as protein active sites. For the same reason, these force fields are marked by low transferability of parameters.

A solution for these issues would be to develop more advanced models that are physics-based and explicitly represent nonadditive effects. Thanks to recent developments of computational hardware, especially graphics processing units (GPU), simulations of these models are currently perfectly feasible on the size and time scales required by protein–ligand systems. Additionally, more robust procedures for fitting analytical expressions to molecular potential surfaces and advanced analytical expressions themselves are available. Thus, a few polarizable force fields are under continuous development, e.g., AMOEBA^18^ or CHARMM-Drude.^19^ Nevertheless, they do not fully meet the expectations in the field of drug design, such as high accuracy, transferability, universality, or productivity. An increasing number of parameters in polarizable FF makes it harder to derive them unambiguously from the available experimental information. Consequently, applications of polarizable force fields to model complex systems, such as protein–ligand complexes, are relatively rarely reported in the literature.^20−23^ Reported ligand-binding free-energy calculations with AMOEBA FF showed lower accuracy^20^ for some systems than calculations with simpler nonpolarizable FF.

An appealing alternative to empirical force fields are force fields that rely purely on QM calculations, e.g., MMFF94,^17^ MB-pol,^24^ QMPFF,^25,26^ QMFF,^27,28^ QMDFF,^29^ or QMPFF3.^26^ Recently, these kinds of force fields have gained traction as accurate but computationally intensive QM calculations, such as those based on coupled-cluster (CCSDT) method, have become more accessible. QM calculations can provide detailed insights into energy interactions within and between the individual particles and also for the individual energy components based on suitable decomposition schemes.^30^ Thus, QM can be used to parameterize advanced physics-based models based on their energy components, e.g., electrostatics, exchange, induction, or dispersion, can be fitted separately. Development of models with terms that have physical interpretation is essential for force field transferability, and high transferability is especially required from QM-based force fields since QM calculations are feasible only for small fragments of compounds.

In this paper, we present results obtained with the ARROW force field. It is an advanced physics-based model that includes multipolar electrostatics and anisotropic polarization. Additionally, its parameters are fitted exclusively to high-level QM data for a set of small compound monomers and dimers, without fitting to any experimental data. We already have shown that ARROW FF provides Δ*G* of solvation for arbitrary neutral molecules with unprecedented accuracy.^31^ Here, we expand the coverage to all standard amino acids (neutral and charged) and limited ligand chemical phase space to make protein–ligand simulations feasible. We probe the accuracy of ARROW FF by calculating the relative binding free energies (ΔΔ*G*) for a series of ligands in MCL1, Thrombin, and CDK2 proteins for which experimental results are known. These systems are also related to real drug design projects and tend to be benchmark studies for various research groups for testing their molecular models.

An equally vital component of any Δ*G* prediction is thorough sampling of configurational space of a simulated system. Successful sampling is especially important for protein–ligand systems that often exist in multiple binding states. Numerous enhanced sampling techniques have been developed to make more states accessible during the molecular dynamics simulations, e.g., umbrella sampling,^32^ TREX (Temperature Replica Exchange), HREX (Hamiltonian Replica Exchange),^33^ REST1, and REST2 (Replica Exchange with Solute Tempering).^34,35^ To ensure adequate conformational sampling, we developed and applied an enhanced sampling technique, a modified HREX coupled to a conformation reservoir generated through softening of the molecular potential, and a nonequilibrium (NEQ) MD.

To successfully disseminate the computational methodology presented in this paper to applications in the pharmaceutical industry, we have developed a user-friendly software package that facilitates ligand parameterization, system setup, running simulations, and their analysis. A key module of the package is ARBALEST,^36^ an MD simulation program that supports the ARROW force field. ARBALEST is capable of running simulations on computer clusters with multiple CPUs and GPUs. It allows a user to perform free-energy calculations and use various enhanced sampling techniques including those described here.

## Theory

### Generation of Conformation Reservoir Using Nonequilibrium MD

Recently, nonequilibrium MD-based techniques were used by several research groups for ΔΔ*G* calculations^37^ and enhanced conformational sampling of ligand-binding complexes.^38^ Free energies for alchemical transitions were calculated from work values computed from multiple nonequilibrium MD runs as the Hamiltonian of the system gradually changes from one state to another in one or both directions.^39,40^ Enhanced sampling on a rugged potential energy landscape of protein–ligand complexes was attempted by Gill et al.^38^ using Nonequilibrium Candidate Monte Carlo (NCMC) moves. NCMC moves consist of nonequilibrium MD runs with the Hamiltonian of the system changing from nonsoftened to a softened state, with an addition of a regular MD stretch in the softened state of the Hamiltonian, followed by a reverse NEQ MD to the nonsoftened state of the Hamiltonian. An enhanced sampling is achieved due to fast interconversions of torsional states of a ligand in MD simulations with “softened” Hamiltonian. NEQ MD work calculations give proper acceptance probabilities for such MC moves that preserve the Boltzmann distribution of molecular system geometries.

In this paper, we use the methodology that can be considered as a modification of the NCMC sampling procedure.^38^ Instead of complex MC moves described above, we are performing nonequilibrium runs from snapshots of equilibrated MD obtained with the softened Hamiltonian, changing the system Hamiltonian from the softened state to the regular (nonsoftened) state. Molecular system geometries at the end of the NEQ MD runs that pass a Metropolis-like acceptance criterion are used to prepare conformation reservoirs for HREX ΔΔ*G* calculations.

First, a sufficiently long MD trajectory with the “softened” Hamiltonian that samples the ligand and protein conformations is generated. The “softened” Hamiltonian is constructed to reduce the potential barriers between the local minima and increase the inter-minima transition rates. For the chosen parameters, 10 ns long MD runs were sufficient to get an equilibrated ensemble of configurations in the “softened” Hamiltonian. To generate a Boltzmann-distributed ensemble of configurations in the original physical (nonsoftened) Hamiltonian, we run a set of nonequilibrium MD simulations starting from a set of geometries in the ensemble of the softened Hamiltonian and filtering the final conformations using a criterion that is based on nonequilibrium work. During the course of a nonequilibrium MD run, the molecular potential quickly changes from the “softened” to “nonsoftened” state. The work for the process is computed as the integral
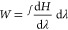
1A Metropolis test is performed for the end point of the NEQ MD trajectory that determines whether to add the end system configuration to the reservoir or not
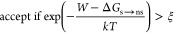
2where ξ is a random number from [0,1] interval and Δ*G*_s→ns_ is the free energy of the transition between softened and nonsoftened Hamiltonians. Δ*G*_s→ns_ is computed iteratively.

The first approximation of Δ*G*_s→ns_ is obtained using the Jarzynski equality
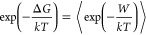
3where ⟨ ⟩ denotes averaging over all of the nonequilibrium MD runs, *W* is the work computed for a nonequilibrium run, thus
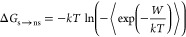
4It is expected that an approximation to Δ*G*_s→ns_ obtained with the Jarzynski equality (eq 4) is not very accurate and a better estimate for Δ*G*_s→ns_ can be obtained using the Maximum-Likelihood method^41^ based on bidirectional nonequilibrium runs, the Bennett acceptance ratio method and the Crooks fluctuation theorem
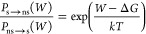
5

In the Maximum-Likelihood method, Δ*G*_s→ns_ is found by solving the equation
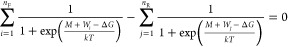
6where  and *n*_F_ and *n*_R_ are the numbers of forward and reverse nonequilibrium MD runs, respectively.

We use values of Δ*G*_s→ns_ obtained in eq 4 to initially filter the end point conformations of NEQ MD runs using the criteria in eq 2. Then, we use these configurations as starting points to run NEQ MD from nonsoftened to the softened Hamiltonian state. Work values obtained in MD runs in both directions are combined, solving eq 6, and the Bennett acceptance ratio (BAR) approach^42^ is used to obtain an improved estimate of Δ*G*_s→ns_ that we plug into eq 2 to obtain an improved equilibrium conformation distribution for the nonsoftened Hamiltonian. The Maximum-Likelihood equation (eq 6) can be solved again to correct values of Δ*G*_s→ns_ based on an updated set of accepted conformations of nonsoftened Hamiltonian. We found that a few iterations were sufficient to converge Δ*G*_s→ns_ to 0.1–0.2 kcal/mol accuracy so that the computed distribution of accepted configurations of nonsoftened Hamiltonian did not significantly change on further iterations.

## Methods

### Quantum Mechanics

In our work, we use a variety of quantum mechanical data as a benchmark for energies and conformations. QM calculations were performed for the monomer model compounds at the MP2/aug-cc-pVQZ level and dimers of the ligand model compounds with amino acid fragments and water were computed with the silver standard, i.e., MP2/CBS, calculated with Helgaker cubic extrapolation from aug-cc-pVTZ->aug-cc-pVQZ as well as post-MP2 correction (i.e., plus CCSD(T)/aug-cc-pVDZ–MP2/aug-cc-pVDZ). More details on QM can be found in the Supporting Information and in our previous publication.^31^

### Force Field

In the ARROW force field, the nonbonded interactions are composed of electrostatic, exchange-repulsion, and dispersion terms. The electrostatic and exchange-repulsion terms are multipolar with inclusion of charges, dipoles, and quadrupoles, and their radial dependence is a Slater-like exponential so that they well describe charge penetration effects. The dispersion term is conventionally represented by spherical terms (C6 and C8), and a Tang–Toennies-damped interaction. Many-body effects are modeled by anisotropic-induced dipoles interacting with the electrostatic and exchange-repulsion terms, as well as with one another. They are iterated to self-consistent field convergence on every nonbonded step. Additional description of the ARROW force field can be found in the SI. For a detailed description, including functional forms, the reader is referred to our previous works.^31,43^

### Parameterization

Proteins and ligands were split into chemical functional groups. Their intermolecular parameters were determined by agreement with QM values of dimer and multimer energies, electrostatic potentials, multipole moments of monomers, polarization tensor, and interaction of fragments with point charges. To aid transferability, we also attempted to match the individual FF energy components to their corresponding QM counterparts, in addition to reproducing the total energy. The typical size of the fragments was not bigger than 10 heavy atoms, e.g., phenol. Larger molecules were built by joining together smaller fragments. We assume that all interactions except electrostatic stay the same (e.g., like in GAFF or AMOEBA) and we refine multipoles on the boundary atoms (e.g., boundary atoms in biphenyl when two benzene rings are merged) to have the best fit to the electrostatic potential around the merged place using the RESP^44^ procedure that is applied to charges, dipoles, and multipoles. For this, we perform QM calculations of lower quality on joined molecular pieces of two fragments. Typically, we include fragments where boundary atoms are either hydrogens attached to carbons or carbons due to their typically more neutral charges in comparison to other more electronegative elements, e.g., oxygen, nitrogen, chlorine, etc. The details of nonbonded parameterization have been described in our previous publication.^31^

### Molecular Dynamics

MD simulations were performed with the ARBALEST simulation package using multiple CPUs with OpenMP and MPI libraries and NVIDIA graphics processing units (GPU) with CUDA library.^45^ For long-range electrostatic interactions, Particle Mesh Ewald (PME)^46,47^ was used. Dispersion and PME direct sum electrostatic interactions were cutoff at 9 Å distance. A multiple time step algorithm was used to integrate the equations of motion.^48^ The system temperature was kept at 298 K using the Nosé–Hoover thermostat.^49^ Pressure was maintained at 1 atm using the Berendsen barostat.^50^

### Systems Setup

The following structures were used to set up three protein–ligand systems for MD simulations: MCL1 (PDB: 4hw2), Thrombin (PDB:2zc9), and CDK2 (PDB:1h1q). Missing residues and side chains in Thrombin were modeled using the Swiss-Model server.^51^ Protonation states of protein residues were determined using PropKa.^52^ Each complex was centered and aligned along its principal axes in a rectangular simulation box. The size of the box was adjusted to leave at least 5 Å distance between the protein and the box edges. The systems were solvated using tools from the GROMACS package.^53^ Some water molecules inserted into the binding pocket were manually removed.

### Systems Equilibration

The solvated protein–ligand systems were equilibrated in two steps. In the first step, all heavy atoms of protein, ligand, and crystallographic water were positionally restrained (*k* = 2.5 kcal/mol/Å^2^). The potential energy of the system was minimized and the system equilibrated for 0.5 ns in the NVT ensemble. In the second step, only the Cα atoms that were farther away than 7 Å from the ligand were restrained. These restraints were maintained in all of the following production and reservoir generation simulations. The energy of the system was minimized again and the system was equilibrated for another 2 ns in the NPT ensemble.

### Free-Energy Calculations

An alchemical transformation method was used for calculations of the free-energy change, Δ*G*_R→T_, associated with mutation of the reference ligand, R, to the target ligand, T. In this method, the Hamiltonian of the reference ligand, *H*_R_, is incrementally transformed to the Hamiltonian of the target ligand, *H*_T_, using a chain of replicas with intermediate hybrid Hamiltonian states, governed by a scalar parameter λ changing from 0 to 1, where 0 corresponds to *H*_R_ and 1 to *H*_T_. The exact coupling relation is described by eq S1 and S2.

The transformations were performed in the protein and solvent to determine Δ*G*_R→T_^protein^ and Δ*G*_R→T_^solvent^, respectively. Free-energy differences, Δ*G*, associated with the alchemical transformations were computed using the Bennett Acceptance Ratio (BAR)^42,54^ and Thermodynamic Integration (TI) methods.^54,55^ Finally, the relative binding free energy, ΔΔ*G*_R→T_, was determined as a difference between Δ*G*_R→T_^protein^ and Δ*G*_R→T_^solvent^.

For asymmetrical ligands for which two values were determined, ΔΔ*G*_A_ and ΔΔ*G*_B_, depending on which site, A or B, the target ligand was modeled into, the following formulas to calculate the combined ΔΔ*G*_A/B_ were used
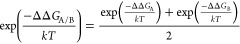
7
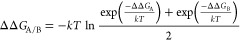
8

### Enhanced Sampling

#### HREX with Potential Softening and Conformation Reservoir

Sampling of the conformational space of replicas used for alchemical transformation was enhanced by the Hamiltonian replica exchange (HREX)^56^ method. In this method, conformations of neighboring replicas periodically exchange, if they fulfill certain energy conditions, increasing the overall sampling. In our simulations, the exchanges are attempted every 120 s. Using real time for the wall time, instead of simulation time, allowed us to efficiently use a cluster of diverse GPUs. A typical alchemical transformation in proteins went through 800 exchange cycles, and 400 exchange cycles in water, roughly corresponding to 2–4 ns.

To efficiently sample conformations of ligands in the protein binding pockets, we reduced the energetic barriers between the potential minima by “softening” selected protein–ligand and ligand–ligand interactions. The softening was introduced into the HREX chain either directly or indirectly via a pre-prepared conformation reservoir. In the direct approach, the softening was gradually turned on from the terminal replicas (λ = 0.0 and λ = 1.0) toward the middle replica (λ = 0.5). As a result, ligands close to the middle were able to sample conformations more efficiently and propagate them toward the terminal nonsoftened replicas through HREX. In the indirect approach, the softening was used to generate a reservoir of enhanced conformations for the reference ligand. Then, the reservoir was coupled to a corresponding replica (λ = 0.0) from which the conformations propagated toward the target ligands (λ = 1.0) through HREX.

Generation of the conformation reservoir consists of two steps. In the first step, a long simulation with softened interactions was performed. In the second step, the softened ensemble was converted to a nonsoftened ensemble, i.e., desired reservoir. We explored two methods for the second step―HREX and NEQ MD. In the HREX approach, the softened ensemble was alchemically transformed to the nonsoftened ensemble by a set of intermediate λ-states (similarly as for the ligand mutation). In the NEQ approach, the Hamiltonian of the softened ensemble quickly changes from the softened to the nonsoftened state. Work and Δ*G* calculated during this process serve to filter a generated nonsoftened ensemble (see the following paragraph for details). Both approaches allow generation of a Boltzmann-distributed ensemble that can periodically insert random conformations to the corresponding replica (here, the reference replica, λ = 0) of mutation HREX.

#### Conformation Reservoir Generation Using Nonequilibrium MD

**Figure 1:**
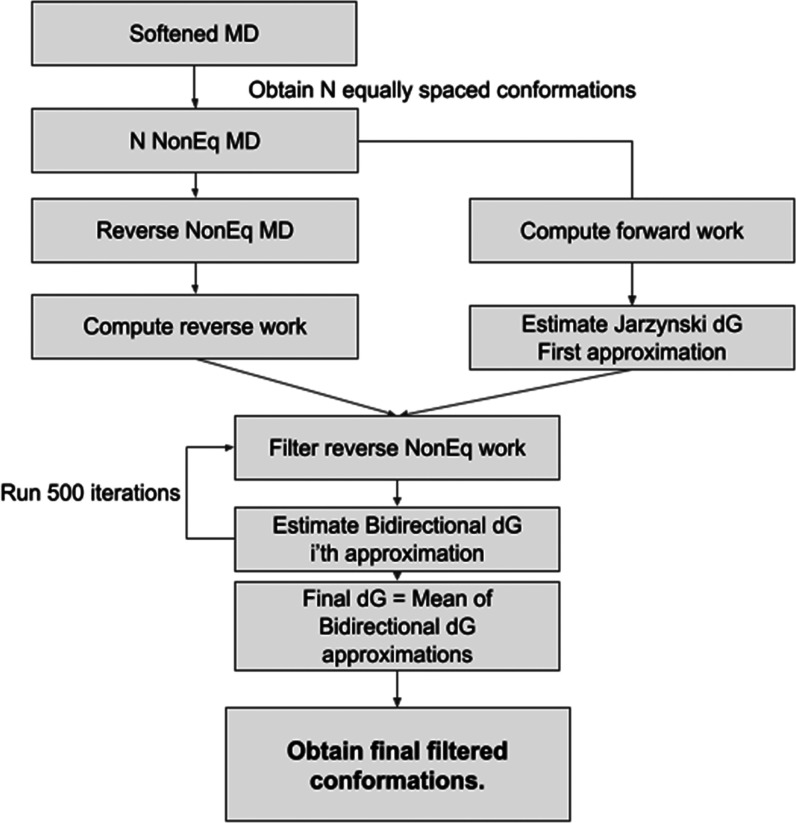
Generation of conformation reservoir using nonequilibrium MD.

In our NEQ MD runs, the Hamiltonian of the molecular system linearly changes from the softened to the nonsoftened state during the time interval *T*, governed by a coupling parameter λ. For the systems studied, it was found that *T* = 10 ps provides a good tradeoff between the computational costs of the simulations and the accuracy of the results. For each of the NEQ MD runs, work values were computed as explained in the Theory section. The starting conformations of NEQ MD runs were drawn from the trajectory generated in MD with a softened Hamiltonian as described above. The following workflow (see Figure 1) was used to generate a conformation reservoir:

(0) Generate the softened trajectory (MD trajectory of the system with “softened” Hamiltonian, having reduced potential barriers between relevant local potential minima of the ligand–protein complex).
(1) Take equally spaced conformations from the softened MD trajectory.
(2) Run NEQ MD starting from the chosen conformations changing the Hamiltonian from the softened to the nonsoftened state (forward NEQ MD runs).
(3) Compute work values for forward NEQ MD simulations, and compute Δ*G* between softened and nonsoftened Hamiltonian states using Jarzynski equality.
(4) Filter the NEQ simulations based on computed work and Δ*G* values using a Metropolis algorithm as described in the Theory section.
(5) Run NEQ MD from the nonsoftened to the softened Hamiltonian state (reverse NEQ MD) starting from the end conformations of filtered forward NEQ MD runs.
(6) Use forward and reverse NEQ MD results to compute Δ*G* with the bidirectional method.
(7) Go to 4.
(8) Repeat steps 4–7 500 times. We need to run reverse NEQ MD only for those configurations that were not filtered in the previous cycles.
(9) Average Δ*G* values obtained in cycles 4–7.
(10) Obtain a final set of filtered MD conformations from forward NEQ runs using the averaged value of Δ*G* between softened and nonsoftened Hamiltonian states (Figure 1).

The methods are described in more detail in the Supporting Information.

## Results and Discussion

### Relative Binding Free-Energy Predictions

**Figure 2:**
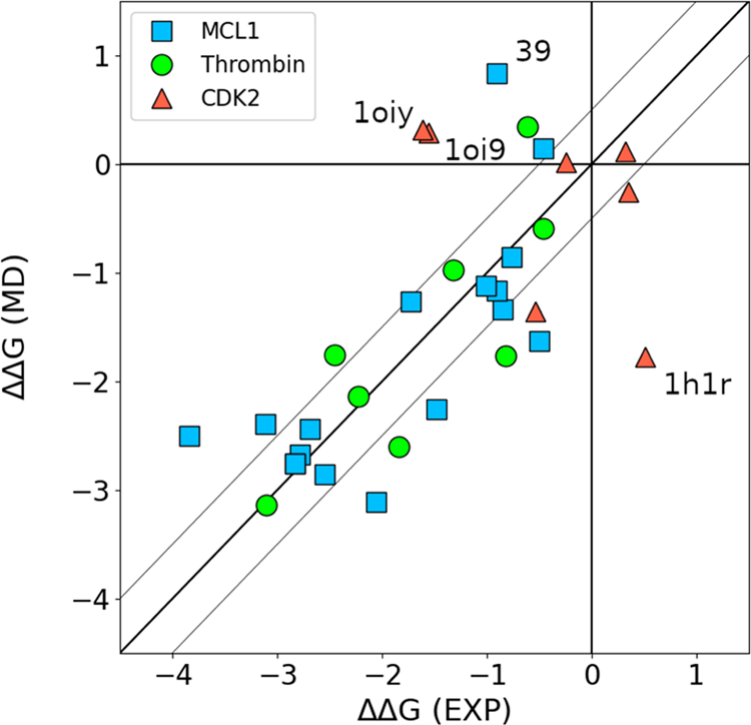
Parity plot comparing the relative binding free energies ΔΔ*G* for ligand mutations in MCL1, Thrombin, and CDK2 as predicted by ARROW FF and the experiment. Results with ARROW FF were calculated with HREX and conformation reservoir generated via potential softening and NEQ MD. Selected ARROW ΔΔ*G* values with the largest deviation from the experiment (MAE > 1.5 kcal/mol) are marked with labels - ligands 1h1r, 1oi9, and 1oiy bound to CDK2, and ligand 39 bound to MCL1. The thin gray lines are +/– 0.5 kcal/mol from the diagonal.

ARROW FF has shown its ability to predict solvation free energy of arbitrary small neutral molecules with unprecedented accuracy (MAE: 0.2–0.3 kcal/mol).^21^ Here, we probe its ability to predict protein–ligand relative binding free energy. Our test set consists of three proteins―MCL1, Thrombin, and CDK2 (Figure S1)―each with a series of binding ligands (Figures S2–S4). The complexes proved to be stable during 10 ns long MD simulations with an average RMSD of Cα atoms from the X-ray structures of 1.3, 1.2, and 1.9 Å for MCL1, Thrombin, and CDK2, respectively (Figure S5). Such a deviation is on a similar level to that of other force fields.^19^ Nonetheless, the following ligand-binding simulations were performed with peripheral Cα atoms positionally restrained to avoid the effects of potential slow conformational changes. The ΔΔ*G* predictions obtained with ARROW FF are shown against experimental values in Figure 2. The exact values, correlation coefficients, slopes, and errors can also be found in Tables S1–S3.

**Figure 3:**
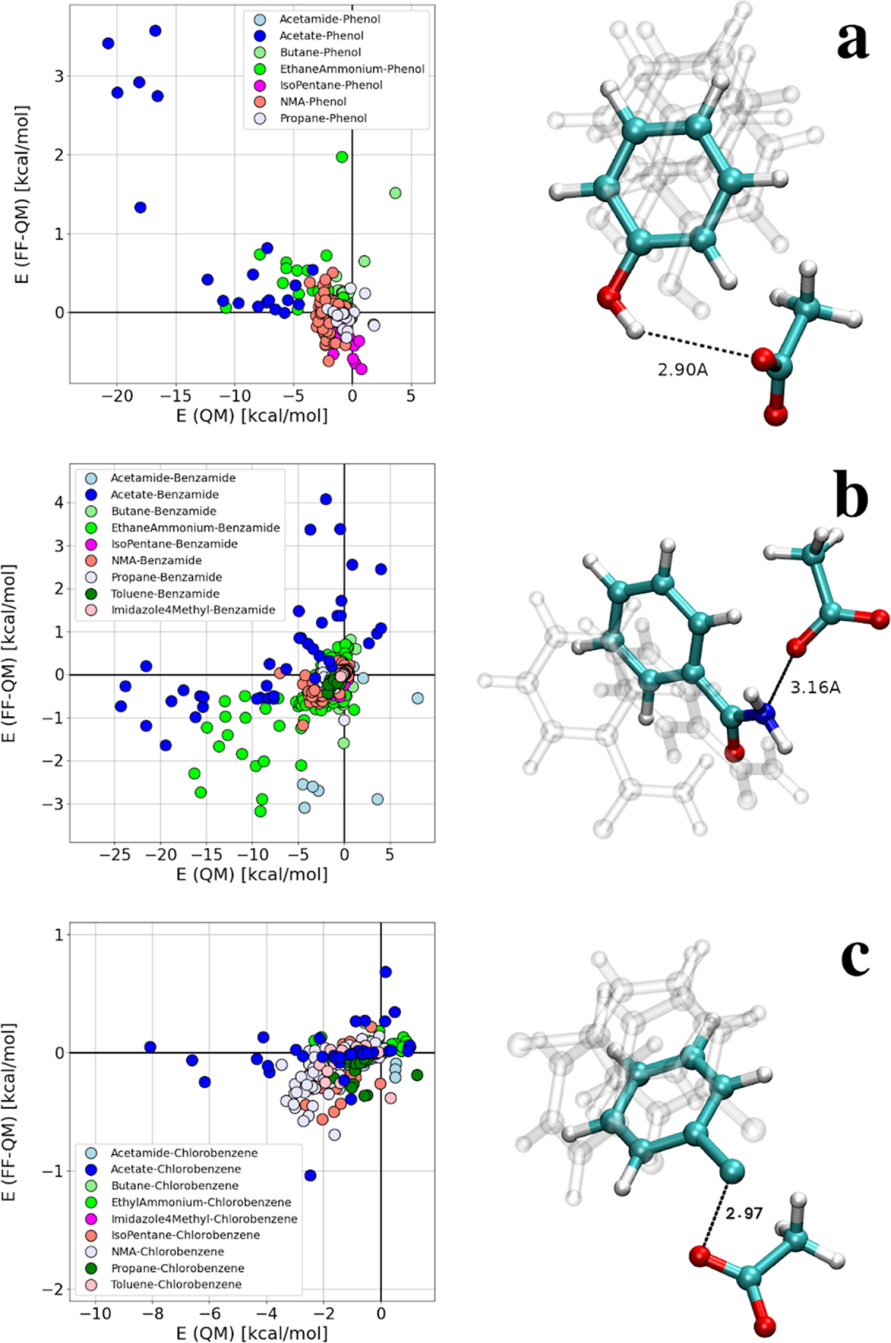
Difference in the interaction energy determined with ARROW FF and QM for amino acid fragments of CDK2 and ligands: (a) 1oi9 (phenol), (b) 1oiy (benzamide), and (c) 1h1r (chlorobenzene). Configurations with the largest discrepancy are shown on the right (opaque) along with a few others (transparent).

Overall, ARROW FF predicts ΔΔ*G*’s well (MAE: 0.7) and at a similar accuracy level as leading nonpolarizable force fields OPLS^10^ (MAE: 0.6), GAFF^9,11^ (MAE: 0.8), and CGenFF (MAE: 0.8)^12^ (see Figure S6 for comparison). Notably, three ΔΔ*G*’s with the largest deviation from the experiment (∼2 kcal/mol) come from mutations in the same protein—CDK2, i.e., mutations of 1h1r, 1oi9, and 1oiy ligands. We analyzed these simulations in detail, looking for putatively incorrectly described protein–ligand interactions and found 1oi9 being the most evident case. Namely, the X-ray structure of 1oi9 in CDK2 (PDB: 1oi9) indicates that a hydroxyl group of the ligand forms a hydrogen bond with the carboxylate group of ASP87. However, such a hydrogen bonding interaction is not observed during the simulations. Lack of a strong O–H···O hydrogen bond well explains the binding being underestimated by ∼2 kcal/mol. Additionally, we found that in simulations with the GAFF force field, this hydrogen bond is present and persists over the entire simulation, ultimately producing ΔΔ*G* with a much smaller deviation from the experiment. To check if the ARROW FF misrepresents this or any other interaction with the 1oi9 ligand, we extracted dimers of protein–ligand fragments from the GAFF simulation and calculated their interaction energy using ARROW FF and QM (“silver standard”). The difference between the two energies, i.e., FF-QM, can be seen in Figure 3a. Indeed, the largest inconsistency is found for a pair of phenol (fragment of 1oi9) and acetate (fragment of ASP87).

Similar FF-QM calculations were performed for ligand 1oiy. Although a hydrogen bond between an amide group of the ligand and a carboxylate group of ASP87 periodically forms during the simulation with ARROW FF, that is consistent with the X-ray structure (PDB: 1oiy), the FF-QM indicates significant discrepancy (Figure 3b). ΔΔ*G* of the third questionable ligand, 1h1r, as opposed to 1oi9 and 1oiy, is overestimated with respect to the experiment. Nevertheless, interactions with ASP87 are likely to be the key in this case too, since the X-ray (PDB: 1h1r) indicates that the chlorine of the ligand and the acetate group of ASP87 are in close proximity (∼3 Å). FF-QM calculations confirm this: finding acetate–chlorobenzene dimers showing the largest discrepancy (Figure 3c). These observations suggest that ARROW FF might not reproduce the interactions that involve charged groups sufficiently well.

It has been shown that for accurate free-energy (Δ*G*) calculations, nuclear quantum effect (NQE) should be taken into account.^43^ However, we found that this effect mostly cancels out in our relative free-energy (ΔΔ*G*) calculations, where Δ*G* determined in water is subtracted from Δ*G* determined in a protein. We repeated two of our calculations of ligands binding to the CDK2 protein with PIMD = 4 (Path Integral Molecular Dynamics), which models NQE. As can be seen in Table S4, although corresponding Δ*G* values are reduced due to NQE, the final ΔΔ*G* values are not very different. For this reason, as well as the high computational cost of using PIMD, we neglected NQE in our present calculations.

### Conformational Sampling

In addition to force field accuracy, adequate conformational sampling is another factor that determines the validity of binding free-energy calculations. Missing or inadequate sampling of strong or weak binding states can result in underestimation or overestimation of the binding free energy, respectively. This question is even more compelling in the case of polarizable models that can significantly raise the bar for the currently available enhanced sampling techniques. Having this in mind, we paid particular attention to extensively sample the conformations in our protein–ligand systems.

For all of the three protein systems studied here, we choose ligands with the simplest benzyl group as a reference. Thus, mutations to the target ligands mostly relied on growing an additional group from the benzyl site. If the group grows asymmetrically, i.e., in *ortho* or *meta* position, then, there are two alternative sites for it (see Figure S7). We call them sites A and B. It is important to mention that in our simulations, any ligand, even the reference, does not flip from site A to B, or vice versa, when it is in the binding pocket. It is unclear if such a flip is possible in reality, or a ligand needs to leave and reenter the packet to do so. In either case, it is unknown apriori how these two sites contribute to the experimental ΔΔ*G*. In the case of 1h1r ligand in CDK2, both the orientations of the chlorobenzene group in *meta* position were resolved from the X-ray experiment (Figure S9). This suggests that each of them should be taken into account in the binding free-energy calculations.

**Figure 4:**
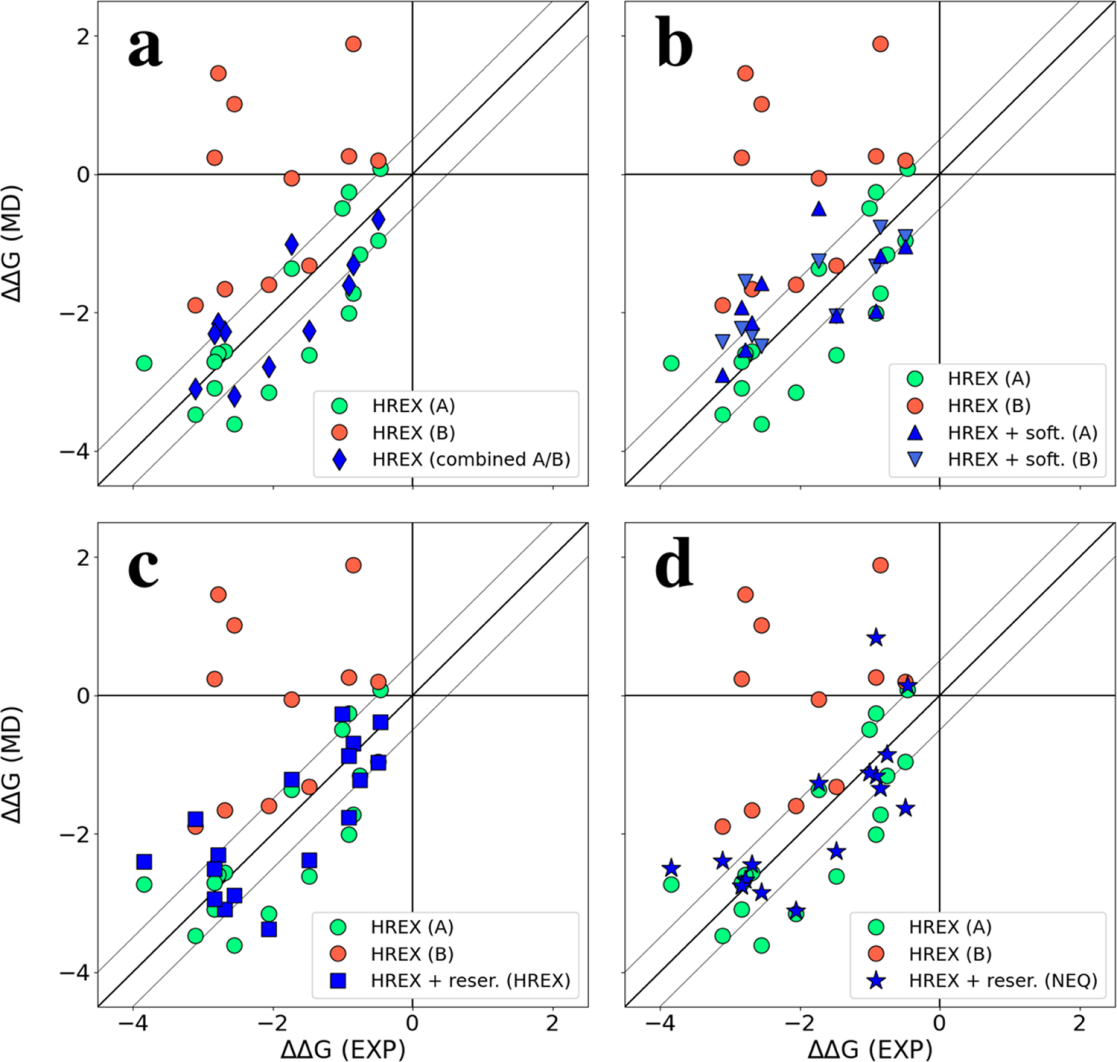
Comparison of ΔΔ*G* as determined with ARROW force field and the experiment for MCL1. Green and red markers correspond to values obtained with HREX and a target ligand starting at A and B sides, respectively. Blue markers correspond to (a) combined A/B sides, (b) HREX with softening from A and B sides, (c) HREX with reservoir (from HREX), and (d) HREX with reservoir (from NEQ). Thin gray lines are +/– 0.5 kcal/mol from the diagonal.

Here, we discuss this sampling problem, and how we deal with it, in detail for the case of MCL1. Indeed, for asymmetrical ligands, we obtained two different sets of ΔΔ*G* values (see Figure 4a) depending on which site, A or B, the ligands started the simulations from (MAE: 0.61/1.38 kcal/mol). Although the obtained Δ*G*’s can be combined according to eq 8, it is in question if there are any more missing states. Furthermore, analyzing the simulations, we noticed that certain torsional angles along the ligand backbone undergo rare transitions. We found that the X-ray structures (PDB: 4hw2, 4hw3) also contain their alternative states (Figure S10). Thus, to sample all of the possible states extensively, including flipping of the benzyl ring, we softened all of the backbone torsions of the ligand (Figure S8a) and also the protein–benzyl interactions. In our first approach, we applied the softening directly to the mutation HREX chain (maximum for the middle replica, λ = 0.5). Although convenient, to keep a sufficient exchange rate between the replicas, we had to increase the number of λ-states from 11 to 21. As expected, the obtained ΔΔ*G* values with this method were mostly found between the A and B states determined with the regular HREX (Figure 4b) (MAE: 0.66/0.49). Nevertheless, they still depend on which site the simulation started from, which is an indication of the convergence issue. Moreover, as the softening applies to the hybrid ligand (λ = 0.5), it might be suspected to not perform as effectively for different mutations.

**Figure 5:**
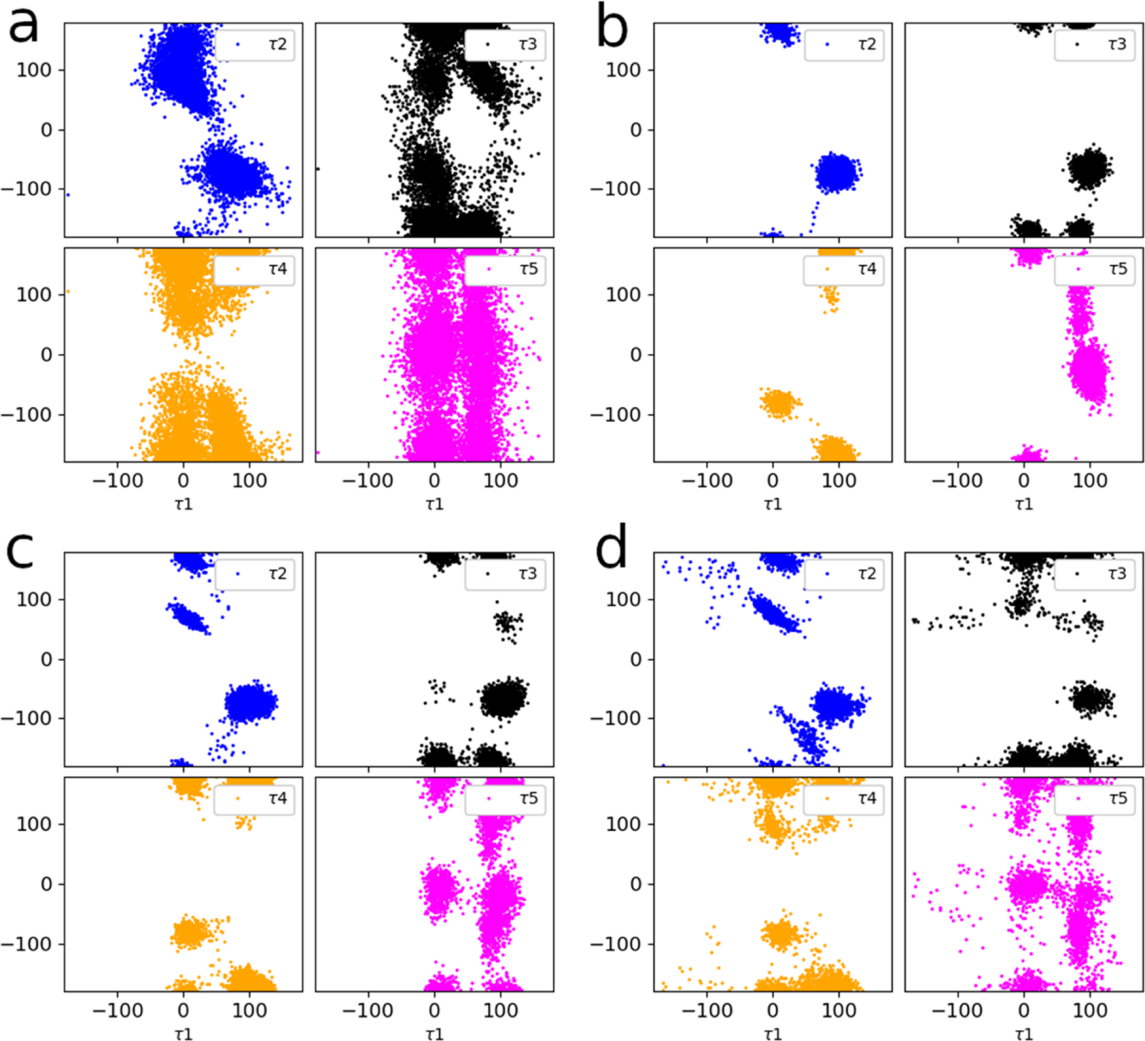
Pairwise distribution of rotatable torsions of ligand 27 in MCL1 from (a) softened potential MD, (b) nonsoftened potential MD, (c) nonsoftened potential MD with a reservoir (generated with HREX), and (d) nonsoftened potential MD with a reservoir (generated with nonequilibrium protocol).

Utilizing our second approach, we deconvolute the softening from the mutation. We run a single long simulation with potential softening of the reference ligand 27 in MCL1. We made sure that all of the torsions and the benzyl ring flipped between the different states multiple times (Figures 5a, S14, and S15). To convert the softened ensemble to the nonsoftened ensemble, i.e., reservoir, we used two different methods—HREX and NEQ. We found that both methods produced similar conformational ensembles (compare Figure 5c,d). What is more, we found that the reservoirs contain conformations otherwise not sampled (compare, e.g., Figure 5d,b). When the reservoirs were attached to the replica of reference ligand 27 (λ = 0.0), the conformations efficiently propagated along the mutation HREX chain enhancing sampling also of the target ligands (see Figure S13 with an example of replica exchanges). ΔΔ*G*’s obtained with both the reservoirs were found consistent with each other as well (compare Figure 4c,d) and either reduced the discrepancy with the experiment (MAE: 0.56/0.59). The only significant difference was noticed for ligand 39 (see Figure 1) whose sampling is particularly challenging because of a large phenyl group. With the NEQ-generated reservoir, ligand 39 was found to partially leave the binding pocket. Nonetheless, because of the high computational parallelizability, the NEQ method was chosen to generate conformational reservoirs for the other systems studied here.

**Figure 6:**
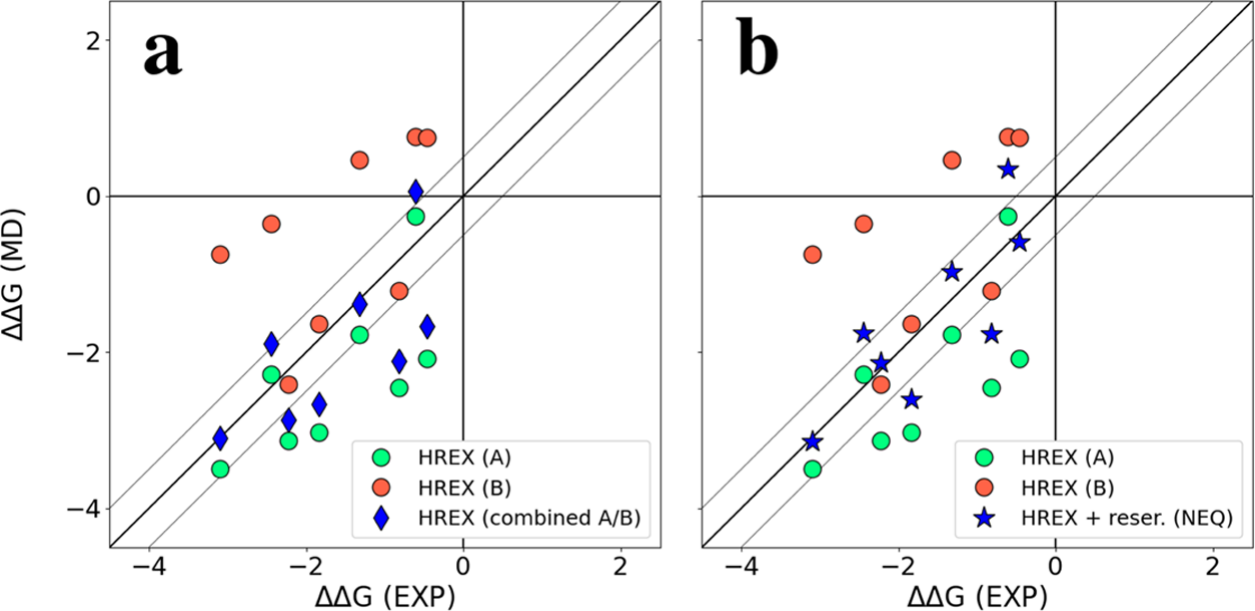
Comparison of ΔΔ*G* as determined with the ARROW force field and experiment (Ki)^57^ for Thrombin. Green and red markers correspond to values obtained with HREX and a target ligand starting at A and B sides. Blue markers correspond to (a) combined A/B sides and (b) HREX with reservoir (from NEQ). The thin gray lines are +/– 0.5 kcal/mol from the diagonal.

Figure 6a shows ΔΔ*G* values determined for a series of ligands binding Thrombin in two alternative orientations, A and B. A-orientation is clearly more preferable than B-orientation and “combined” ΔΔ*G* (A/B) values computed with eq 8 values are very close for A-side ΔΔ*G*’s and agree well with the Ki experiment (fluorescence labeling) (MAE: 0.66). Nevertheless, coupling of HREX with a conformation reservoir generated using nonequilibrium MD makes the predictions even more accurate (Figure 6b, MAE: 0.50).

### Nonequilibrium MD

**Figure 7:**
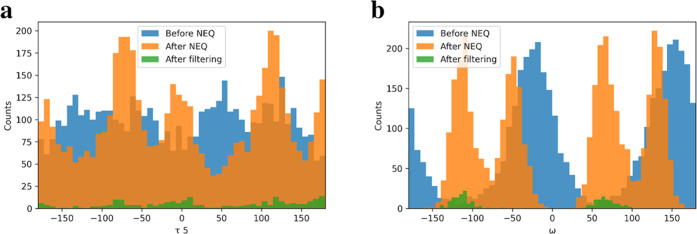
Distributions of benzyl ring (a) torsion τ5 for ligand 27 bound to MCL1 and (b) torsion ω of ligand 5 bound to Thrombin before and after NEQ MD runs for conformer reservoir generation.

To generate a Boltzmann-distributed reservoir of conformation with regular, i.e., nonsoftened, Hamiltonian, conformations from the trajectory with “softened” protein–ligand interactions were used as starting points for nonequilibrium MD runs. Figure 7a shows the distribution of the torsion angle τ5 (see Figure S8a) that describes the orientation of the benzyl ring of ligand 27 in MCL1—before NEQ MD runs (the softened MD trajectory), at the end of 10 ps NEQ MD runs, and after filtering based on the computed work as described in the Theory section. Figure 7b shows the same distributions for the torsion angle ω (see Figure S8b) describing the orientation of the benzyl ring of ligand 5 in Thrombin. One can see how NEQ MD runs and filtering change the observed distributions of benzyl torsions. Especially, it can be clearly seen for ligand 5 of Thrombin (Figure 7b). For the regular nonsoftened Hamiltonian, the distribution of the benzyl torsion angle ω has narrow peaks around −120 and 60°. Distribution of ω angle for softened Hamiltonian MD before NEQ MD runs is wide with probability maxima around −20 and 160°. After NEQ MD runs, the distribution of ω angle shows four narrow peaks at −120, −60, 60, and 130°. NEQ work-based filtering removes configurations at −60 and 130° so filtered configurations have the correct Boltzmann distribution corresponding to the nonsoftened Hamiltonian of the system.

As outlined in the Methods section, bidirectional Δ*G* was computed via the iterative procedure. The value Δ*G* convergence is defined here as Δ*G* (bidirectional). In two systems studied, free energies computed via the bidirectional approach and Jarzynski equality differ. For MCL1 ligand 27: Δ*G* (Jarzynski) = −34.20 kcal/mol, Δ*G* (bidirectional) = −32.26 kcal/mol. For Thrombin ligand 5: Δ*G* (Jarzynski) = −2.20 kcal/mol, Δ*G* (bidirectional) = −1.83 kcal/mol. It is known that Jarzynski equality expression is strongly affected by tails of the work distributions often resulting in too negative computed Δ*G* values. The bidirectional approach is not prone to these problems and provides more robust estimates of Δ*G* between the softened and nonsoftened states.

**Figure 8:**
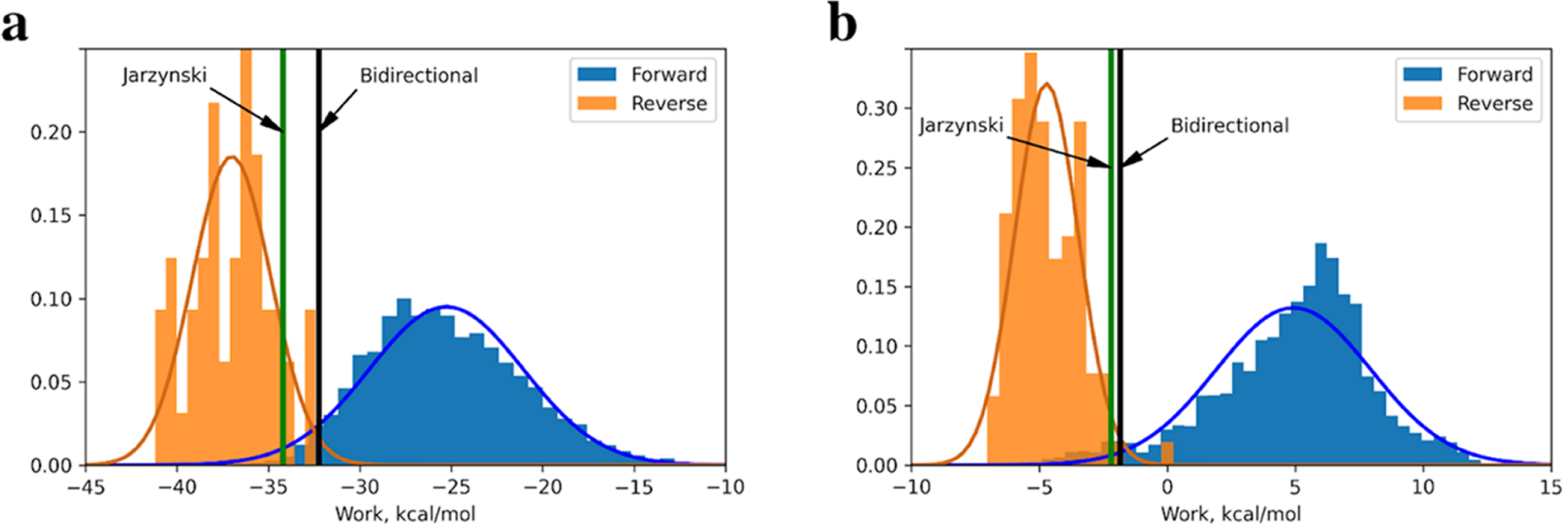
Forward and reverse work distribution for (a) MCL1 ligand 27 and (b) Thrombin ligand 5. Δ*G* values between softened and nonsoftened Hamiltonian states of the system computed with one-directional (Jarzynski) and bidirectional (Crooks theorem-based) approaches are shown with green and black vertical lines.

Figure 8 shows distributions of work values computed for forward and reverse NEQ MD runs. The distribution of reverse work is sparse since the reverse NEQ MD starts from end conformations of forward NEQ runs that passed the Metropolis criteria (see Theory Section). The acceptance ratio for the MCL1 ligand 27 system was 4.8% and that for Thrombin ligand 5 was 4.7%. Blue and orange curves represent Gaussian curves that fit distributions of forward and reverse work values, respectively. The crossing point of these curves may be used as another estimate of the free-energy difference between softened and nonsoftened states of the molecular system and is close to the estimate obtained by the bidirectional approach. Green and black vertical lines correspond to the Δ*G* values computed via Jarzynski and bidirectional approach.

## Conclusions

Based on the three protein–ligand systems, we have shown that ARROW FF is able to predict relative binding free energy with almost chemical accuracy and is currently on par with leading all-atom fixed-charge force fields. We identified that the largest discrepancy with the experimental results is associated with binding interactions that involve charged groups. Analogous QM simulations demonstrated that these kinds of interactions are not well represented by the model and will be addressed in a later publication.

Despite current limitations, ARROW proves its potential in the field of drug design. As our model is physics-based and relies purely on QM calculations, the sources of error can be narrowed down to particular energy terms and refined separately. Additionally, because our technology does not require any experimental data, the force field refinement can be performed in a systematic manner. Although, in general, this is an endless process, we believe that there is a particular complexity and accuracy of the model that needs to be reached for successful drug design. We think that making a force field faithful to high-level ab initio calculations is a step in that direction.

Since effective sampling is a common challenge and usually cannot be completely deconvoluted from the force field accuracy, here, we paid particular attention to sufficiently sample protein–ligand conformations. We have shown that a conformation reservoir generated through potential softening in a nonequilibrium process is an efficient way to extend conformational space of a ligand in the protein binding pocket.

## Supporting Information Available

Details of the methods, comparison of the enhanced sampling techniques, comment on alternative experimental results for Thrombin, structures of proteins and ligands, tables with values of the binding free energy, comparison with other force fields, and RMSD of the proteins (PDF)

## Notes

InterX Inc. is a subsidiary of NeoTX Holdings Ltd., Rehovot, Israel 7670202.
