# Supplementary material for: Protein–Ligand Binding Free-Energy Calculations with ARROW—A Purely First-Principles Parameterized Polarizable Force Field
Source: J Chem Theory Comput. 2022 Dec 2;18(12):7751–63. doi: 10.1021/acs.jctc.2c00930 (PMC9753910; doi:10.1021/acs.jctc.2c00930)
Supplement: Supplementary file 1 — ct2c00930_si_001.pdf [file ct2c00930_si_001.pdf]

Supporting Information

Protein-ligand binding free energy calculations with ARROW - a purely first principle parameterized polarizable force field

Grzegorz Nawrocki[1*], Igor Leontyev[1], Serzhan Sakipov[1], Mikhail Darkhovskiy[1], Igor Kurnikov[1], Leonid Pereyaslavets[1], Ganesh Kamath[1], Ekaterina Voronina[1,2], Oleg Butin[1], Alexey Illarionov[1], Michael Olevanov[2], Alexander Kostikov[1], Ilya Ivahnenko[1], Yevhen K. Cherniavskyi[1], Dhilon S. Patel[3], Subramanian K. R. S. Sankaranarayanan[4,5], Maria G. Kurnikova[3], Christopher Lock[1,6], Gavin E. Crooks[1], Michael Levitt[7], Roger D. Kornberg[7], Boris Fain[1*]

[1] InterX Inc., Berkeley, California 94710, USA, a subsidiary of NeoTX Holdings Ltd., Rehovot, Israel 7670202
[2] Faculty of Physics, Lomonosov Moscow State University, Moscow 119991, Russia
[3] Department of Chemistry, Carnegie Mellon University, Pittsburgh, PA 15213, USA
[4] Center for Nanoscale Materials, Argonne National Lab, Lemont, IL 60439, USA
[5] Department of Mechanical and Industrial Engineering, University of Illinois, Chicago, IL 60607, USA
[6] Department of Neurology and Neurological Sciences, Stanford University School of Medicine, Palo Alto, CA 94304, USA
[7] Department of Structural Biology, Stanford University School of Medicine, Stanford, CA 94305, USA
* Corresponding authors: boris.fain@interxinc.com, grzegorz.nawrocki@interxinc.com

METHODS

Quantum Mechanical details

In our work we use a variety of quantum mechanical data. For monomer fragments used for intermolecular parameterization we use the following: an electrostatic potential map with a large number of points on many vdw-distances from the molecule (thousands per fragment), dipoles, quadrupoles, and a polarization tensor as well as energies of interaction (hundreds per heavy atom in the fragment) with a small probe charge (typically 0.1 proton charge). All of these calculations are performed at the MP2/aug-cc-pVQZ level. Such monomer fragments cover the chemical space of proteins and simulated ligands. For merging several fragments we calculate electrostatic potential on a less expensive level of MP2/aug-cc-pVTZ(d/p).

For dimer energy we use high quality QM data because small inaccuracies from these can translate into large errors when typical fragments of interactions are repeated, e.g. N-methyl acetamide with water. We use total energies calculated with the silver standard *i.e.* MP2/CBS, calculated with Helgaker cubic extrapolation from aug-cc-pVTZ->aug-cc-pVQZ as well as

post-MP2 correction (i.e. plus CCSD(T)/aug-cc-pVDZ - MP2/aug-cc-pVDZ). To improve transferability and facilitate optimization we use DFT-SAPT decomposition with dHF correction at aug-cc-pVTZ level with PBE0 functional. We employ four parts of DFT-SAPT decomposition which have corresponding manifestation in our FF : ES (electrostatic E1pol), EX (exchange-repulsion E1exch), IND (induction, E2ind + E2ind-exch + δHF), DS (dispersion, E2disp+E2disp-exch + Esilver-standard - DFT-SAPTtotal_energy). The dispersion term accumulates all disagreements between total energy described by “silver standard” and DFT-SAPT+δHF energies. Further details on QM calculations for the dimers can be found in the Supplementary methods in our previous publication [1]. These dimers include interaction between protein fragments, between protein fragments and ligand fragments, and between protein and ligand fragments and water.

The diversity of ligand chemical space requires special accuracy in bonded space. Every unique typified bond, angle, bond-angle and out-of-plane perturbation of small fragments of ligand were performed from the ground energy state of the ligand. Correct torsional perturbations play a key role in a configurational space of ligand molecules. We sample torsion energies with rigid rotational perturbations as well as “relaxed” perturbation where coordinates of all atoms are optimized except for ones in a particular fixed torsion of interest. In rare cases we have performed two dimensional rigid and relaxed potential energy scans. In cases where the ligand was larger than 18 heavy atoms, it was split into smaller pieces and hydrogens were added in the place of covalent bonds removed. All QM calculations for bonded perturbation were performed at MP2/aug-cc-pVTZ level.

Force field description and parameterization strategy

We are developing a force field that is based on the best available high accuracy QM data and a complex physics-based functional form. In our development we rely on previous discoveries in force fields. Our model - the ARROW Force Field or ARROW FF - has the following features: The non-bonded interactions are composed of electrostatic, exchange-repulsion and dispersion terms. The electrostatic and exchange-repulsion terms are multipolar with inclusion of charges, dipoles and quadrupoles, and their radial dependence is a Slater-like exponential so that these are better able to describe charge penetration. The dispersion component is conventionally represented by spherical terms (C6 and C8), and a Tang-Toennies-damped interaction. Many-body effects are modeled by anisotropic atomic induced dipoles interacting with the electrostatic and exchange-repulsion terms and with each other and iterated to self-consistent field (SCF) convergence on every non-bonded step. The intermolecular parameters of ARROW FF are determined by agreements with QM values of dimer and multimer energies, electrostatic potentials, multipole moments of monomers, polarization tensor and interaction of fragments with point charges. To aid transferability, we also attempt to match the individual FF energy components to their corresponding QM counterparts, in addition to reproducing the total energy. We use the functional form of the bonded interactions that is taken from MMFF94, with force constants and equilibrium values fitted to QM energies. The functional form details can be found in our previous works [1,2] which in turn are based on the QMPFF3 force field functional form [3,4].

We assign force field types based on our knowledge of different chemical functional groups. This is somewhat correlated with the MMFF94 approach. Every atom that is different under topology from another has its own type in every chemical functional group. Various aromatic or heterogeneous rings are considered as independent chemical functional groups. Sometimes force field types in different functional groups are merged by their chemical similarities, e.g. carbonyl carbon and oxygen in acetamide and N-methyl acetamide.

We split protein and ligands into chemical functional groups and parameterize them using monomer, dimer quantum mechanical data as described above. Typical size of the fragments is not bigger than 7 heavy atoms, e.g. phenol. Bigger molecules are built joining together smaller fragments and applying special rules that modify electrostatic parameters of "joint" atoms at the interface between fragments. The details of non-bonded parameterization have been described in supplementary information to our previous publication [1]. The interaction between neutral fragments typically has MAE on the order of ~0.3 kcal/mol (comparable to our previous work, where one of the two fragments was a water molecule), while the error is bigger (MAE about ~1 kcal/mol or worse) for charged-neutral fragments interactions. There are many factors that explain the origin of errors for charged groups.

First of all, the interaction energies of the charge with the neutral species (dimers) is large (e.g. about -20 kcal/mol), if not the largest for the entire set of interactions present in the protein-ligand complexes. These interaction energies are also large in comparison to a typical hydrogen bond (e.g. water-water) interaction which is about -5 kcal/mol. To further complicate the issue, the minimum and its corresponding potential energy surface for these charged-polar interactions is much narrower and harder to describe with the same accuracy as neutral compounds using the functional form. The second reason is that despite the functional form being sophisticated, it is unable to provide an equally accurate description of interactions for all the species so the scale of errors tends to be larger; the errors being roughly proportionally bigger. While our force field is able to predict neutral-neutral hydrogen bonded interactions with a MAE of about ~ 0.3 kcal/mol, our charge-neutral interactions tend to be proportionally worse and the MAE for such interactions is about ~1 kcal/mol or bigger, see e.g. **Figure 3**. Our functional form has physical terms that are proportional to the overlap integral *i.e.* exchange-repulsion and part of induction. It was shown that this also covers energy of charge transfer which is proportional to overlap integral. We believe that the complexity and magnitude of interactions as well as limitations of the functional form to the applied force field is the main reason for imperfect description of the potential energy surface, and not specific interactions such as "charge transfer". The errors are larger for every part of the interactions, e.g. electrostatics, penetration energy, exchange-repulsion, dispersion, and induction, as these occur at a bigger overlap due to stronger interactions.

Alchemical ligand transformations

The alchemical ligand transformation method was used for calculations of relative binding free energies (RBFE). In this method the Hamiltonian of the reference ligand (λ=0) is

incrementally transformed to the Hamiltonian of the target ligand using a chain of intermediate hybrid Hamiltonian states (λ-states), governed by a scalar parameter λ.

During the alchemical transformation, atoms of the hybrid ligand molecule with a common topology, referred to as the COMMON part, are mutated directly from one to another. Within the COMMON part all the interactions (bonded and non-bonded) are coupled between the A (λ=0) and B (λ=1) states linearly:

$$U_{\lambda} = (1 - \lambda)U_{A}^{Com}(r) + \lambda U_{B}^{Com}(r) \qquad \text{(Eq. S1)}$$

Topologically distinct groups of the hybrid molecule are transformed to corresponding DUMMY parts. Bonded interactions of the DUMMY atoms with the COMMON part atoms are coupled linearly based on **Eq. S1**. Non-bonded interactions of the DUMMY atoms with the COMMON part or with atoms of any other DUMMY part are switched between the "real" and "dummy" states with a nonlinear soft-cored function:

$$U_{\lambda} = (1 - \lambda)^{k}U_{A}^{Dum}(r + \lambda r_{sc}n) + \lambda^{k}U_{B}^{Dum}(r + (1 - \lambda)r_{sc}n) \qquad \text{(Eq. S2)}$$

where r_{sc} is the soft-coring radius parameter, k is the scaling factor power and n is the unit vector of the vector r. In the alchemical pathway, we decouple both the electrostatic and van-der-Waals interactions simultaneously. The soft-core **Eq. S2** helps to avoid singularities and instabilities in intermediate lambda states. The soft-coring radius r_{sc}=1.5 Å and the scaling factor power k=2 provided smooth shape of the dG/dλ profiles, lower statistical errors and no noticeable sampling-trapping artifacts for all studied compounds.

As λ changes from 0 to 1, some ligand-protein and ligand-ligand interactions are gradually turned on and others are turned off. The mixed single and dual topologies approach [5] was used to specify alchemical ligand Hamiltonian transformations. The COMMON part of the hybrid molecule for most of the compounds was defined as the maximum common substructure (MCS) between the reference and target compounds while the rest was defined as a DUMMY part. Typically we used 11 replicas spanning λ from 0.0 to 1.0 with an interval of 0.1. For some mutations it was beneficial to add two extra states: λ=0.05 and 0.95 totaling to 13 λ-states to provide a finer description of the alchemical transformation.

In the cases where a rotation around the bond next to the DUMMY-COMMON linker bond was important for conformation sampling, the COMMON part was reduced by one heavy atom in order to provide scaling down of the high barrier torsion potential in the course of the mutation, and achieve thermal accessibility of the important rotameric states in the HREX simulations.

Here, we give the details about how the bonded interactions involving the dummy atoms are treated in free energy perturbation (FEP) to avoid singularities and instabilities. It is known

that if the dummy atom does not have any bonded interactions with the rest of the molecule (COMMON part) and moves freely in the whole simulation volume it makes the sampling very difficult. Thus, in the molecule end state ARBALEST keeps the dummy atoms bonded with the rest of the molecule. However, if there is more than one bonded stretch or bonded angle or bonded dihedral angle interactions between a dummy atom and the rest of the molecule, the distributions sampled for the hybrid molecule (molecule including the dummy atoms) will be different from the molecule without the dummy atoms. So ARBALEST keeps only one bonded stretch, bonded angle, and bonded dihedral angle interactions involving a dummy atom whereas all the other bonded interactions are scaled to 0 at the end state. In this way, the contributions of the dummy atoms to the free energies in the binding complex and in pure solvent will be identical and cancel out in the relative binding energy providing its independence from the choice of dummy atoms.

For most systems, this method works well, but for some systems with certain symmetry, it will cause serious problems in the FEP simulation. To avoid possible instabilities and singularities caused by the missing bonded interactions involving dummy atoms, the remaining dihedral potential in the end state is transformed to the single minimum function that keeps dummy atoms at the initial state position. This ensures that in the end state the dummy atom will keep its position as in the initial state and does not switch to the position of other atoms of the COMMON part that results in clashes and instabilities. Although the distributions sampled for the hybrid molecule might be a bit different from the distributions for the molecule without the dummy atoms, the error introduced in this treatment is negligible because the fluctuations of bond angle and transformed dihedral are sufficiently small for the sets of ligands studied in this article. In addition, the error in the relative binding affinity becomes even smaller due to cancellation of errors between the free energies of the protein-ligand complex and ligands in the solvent.

For each λ-state an MD simulation of the molecular system is performed. Alchemical transformations were performed independently in protein and water environments. The relative binding free energy was calculated as a transformation free energy in a protein minus the transformation free energy in water. Statistical sampling of the molecular system conformations was enhanced by Hamiltonian replica exchange (HREX)[6] between the neighboring λ-states. Our typical alchemical transformation simulation went though 1,200 exchange cycles. Replica exchanges were attempted every 120 seconds in wall time, thus, the actual simulation time of each λ-replica varied. This strategy allowed us to efficiently use a cluster of diverse GPU's.

Enhanced sampling

HREX with potential softening

In order to compute relative free energies of ligand binding we performed simulations of alchemical transformation of ligands in protein and in water with conformational sampling enhanced by Hamiltonian Replica Exchange in the space of λ-replicas (HREX) [6]. The molecular Hamiltonian in the middle λ points is "softened" so the potential barriers between the

conformations of the ligand and surrounding protein residues are lowered and rates of transitions between the local potential minima are greatly increased. Conformations generated in the middle λ-replicas are propagated to the end points of the transition as a result of HREX, thus, after certain simulation time Boltzmann equilibrium distribution is established for the ligand and protein conformations and for all λ points of the transition. This approach is similar to the REST2 method.[7]

Disadvantages of this in-the-middle potential softening approach compared to regular λ-HREX simulations include the increase of the number of intermediate λ-states needed to ensure a sufficient acceptance rate of replica exchanges. The greater number of atoms involved in the softening of the Hamiltonian and greater the change in the Hamiltonian the more replicas are needed in alchemical HREX simulations. The scale of changes of dG/dλ values as a function of λ is also increased with an increased potential softening at middle λ values. This results in a larger statistical uncertainty of the computed ΔG values and longer MD simulation time needed for ΔG convergence. The larger number of λ-states also means a slower propagation of system conformations due to replica exchange from the middle to the end λ-states again resulting in a slower ΔG convergence.

HREX with conformation reservoir

Alternatively, the statistical sampling of protein-ligand geometries during the alchemical transformation was enhanced by attachment of a reservoir of conformations of the simulated system to the λ-HREX chain. The reservoirs were prepared beforehand and contained conformations from local potential minima of the ligand-protein distributed with Boltzmann probabilities. Random conformations from the reservoir were inserted every two exchange cycles to the λ=0 replica and were allowed to propagate along the chain of λ-replicas using HREX. The reservoir could be generated only once since the same reference ligand was used for a full series of mutations. Conformation reservoirs were prepared in two steps. In the first step a widely sampled conformational ensemble was generated in a single MD simulation by softening particular protein-ligand and ligand ligand interactions. In the second step the "softened" ensemble was converted to the "non-softened" ensemble using either HREX or non-equilibrium approach.

MD with softened potential

In order to efficiently sample conformations of ligands in the protein binding pockets we reduced the energetic barriers between potential minima by "softening" particular ligand-ligand and ligand-protein interactions. For ligand 27 in MCL1 torsion potentials along the ligand backbone (τ1-5, see **Figure S8a**) were scaled by a factor of 0.3. The non-bonded interactions within the ligand were scaled by a factor of 0.1 and soft-cored using the radius of 0.5 Å. Additionally, to allow the benzyl group to rotate, non-bonded interactions between this group and the protein were scaled by a factor of 0.1 and soft-cored using the radius of 1.5 Å. A molecular dynamics simulation with a potential softening of ligand 27 in MCL1 was run for 10 ns. 500 conformations were extracted from this trajectory using a 20 ps interval which further served as a softened ensemble.

Conformation reservoir generation using HREX

HREX was applied to generate a non-softened reservoir of conformations from the softened ensemble defined above. 17 replicas with the Hamiltonian spanning the softened (λ=0) to the non-softened (λ=1) potential were used. Random conformations from the softened ensemble were inserted into the softened replica (λ=1) every two exchange cycles, from where they had a chance to propagate toward the non-softened replica (λ=0) if favorable. 3,200 exchange cycles were performed. After skipping the first 1 ns as an equilibration the conformations sampled by the non-softened replica (λ=0) were extracted every 10 ps to generate a reservoir for the free energy calculations. The reservoir for mutations in MCL1 contained 683 geometries of ligand 27 - MCL1 complex, covering the accessible conformational space.

RESULTS

Enhanced sampling techniques comparison

We have shown that three enhanced sampling techniques, i.e. in-the-middle softening, HREX reservoir, and non-equilibrium reservoir, improve ΔΔG predictions for MCL1. Each of these allows sampling of ligand conformations otherwise omitted in the regular MD. The advantage of the in-the-middle softening method is its simplicity, since the enhanced sampling is a part of the mutation λ-HREX chain. However, it is also a weakness of this method, since maximum softening applies to the hybrid system (λ=0.5, mix of half grown reference and target ligands), thus, optimal softening parameters might differ between mutations. Additionally, ΔΔG convergence requires more λ-replicas and longer MD runs. As we have shown, 17/21 replicas simulated over roughly 3-6 ns (1,200 exchange attempts) do not guarantee stable results.

Application of the other two enhanced sampling techniques is more complex since it requires an additional step - generation of a conformation reservoir. For either HREX or non-equilibrium reservoir a softened trajectory needs to be generated beforehand. It can be considered as a “bottleneck” of these methods since selection and adjustment of the softening parameters is tricky. The softening should favor the same, or at least similar, important states as the regular Hamiltonian. On one hand, it should be sufficient to allow an easy transition between these states. On the other hand, softening should not be exaggerated, as a large phase space of available states can make the generation of a Boltzmann distributed ensemble impossible. Then, the softened ensemble needs to be converted to the non-softened ensemble, i.e. the reservoir, by either λ-HREX chain or non-equilibrium process. We have shown for MCL1 that conformation reservoirs produced with the both approaches are similar and the ΔΔG values obtained with them are close. However, the advantage of the non-equilibrium over the HREX method is that it can make use of a large number of diverse computational resources. In an extreme case, all the non-equilibrium processes can be run independently at once, thus, the overall performance depends only on the performance of a single process. In our study, generation of a reservoir that consists of 230 conformations of ligand 27 in MCL1 required

running of up to 3,900 non-equilibrium runs, 10 ps each (total MD length is ~40ns). In contrast, the HREX method needs parallel computing resources limited to the number of replicas, all run long enough to reach the overall equilibrium. In our study, 17 replicas of ligand 27 in MCL1 were run over 9.3-19.0 ns with 3,200 exchange attempts (total MD length is ~250ns). Although reservoir generation requires additional effort, it needs to be performed only once, for the reference ligand, as in our study, and new conformations can propagate efficiently to any target ligand along the mutation λ-HREX chain. Without in-the-middle softening only 11 λ-states were needed in the alchemical transitions for efficient ΔG integration and fast replica exchange. 4-6 ns MD runs for λ-states were used for converged results in HREX with reservoir calculations (total MD length ~50 ns per mutation), while computational cost for in-the-middle softening calculations were about doubled per mutation (~ 100 ns) and ΔΔG was still not fully converged.

Alternative experimental results for Thrombin

Figure S11 shows the computed ΔΔG values in Thrombin against alternative experimental results from isothermal titration calorimetry (ITC). Both series correlate well (r=0.96), however, the slope of the linear correlation is far from unity (slope=1.90). The correlation of computed results to the other set of binding ΔΔG experimental data reported in the same paper based on the fluorescence measurements of ligand substitution in the binding pocket (K_i) is somewhat worse (r=0.81), while the slope of linear fit of theoretical ΔΔG values to experimental ones is closer to unity (slope=0.96) (**Figure 5**). It is likely that experimental ITC data had a systematic error that resulted in a smaller linear correlation slope between experimental and theoretically computed ΔΔG values.

We found that stability of the ligand bound conformations and ΔΔG values of mutations in Thrombin are very sensitive to torsional parameters of the ligands. When ligand torsional parameters were fit to QM calculations the ligand was not completely stable in the protein pocket during long MD simulations. Therefore, in ΔΔG calculations we applied positional restraints to the part of the ligand after the C-N bond in the amide group of the ligand (see **Figure S8b**).

Enhanced sampling for CDK2

ΔΔG calculations with ARROW FF were performed for seven ligands bound to CDK2 protein using HREX simulations (no additional softening and reservoirs) starting from two DUMMY orientations, A and B, of the benzene ring of 1q1h ligand. Some “combined” A/B ΔΔG values were markedly different from the experimental values (**Figure S12a**). The attachment of the conformation reservoir obtained by NEQ MD runs did not change the computed ΔΔG significantly (**Figure S12b**). Thus, we conclude that insufficient sampling is an unlikely reason for deviation of theory from experiment in this case.

REFERENCES

(1) Pereyaslavets, L.; Kamath, G.; Butin, O.; Illarionov, A.; Olevanov, M.; Kurnikov, I.; Sakipov, S.; Leontyev, I.; Voronina, E.; Gannon, T.; Nawrocki, G.; Darkhovskiy, M.; Ivahnenko, I.;

Kostikov, A.; Scaranto, J.; Kurnikova, M. G.; Banik, S.; Chan, H.; Sternberg, M. G.; Sankaranarayanan, S. K. R. S.; Crawford, B.; Potoff, J.; Levitt, M.; Kornberg, R. D.; Fain, B. Accurate Determination of Solvation Free Energies of Neutral Organic Compounds from First Principles. *Nat. Commun.* **2022**, *13* (1), 414.
(2) Pereyaslavets, L.; Kurnikov, I.; Kamath, G.; Butin, O.; Illarionov, A.; Leontyev, I.; Olevanov, M.; Levitt, M.; Kornberg, R. D.; Fain, B. On the Importance of Accounting for Nuclear Quantum Effects in Ab Initio Calibrated Force Fields in Biological Simulations. *Proc. Natl. Acad. Sci. U. S. A.* **2018**, *115* (36), 8878–8882.
(3) Donchev, A. G.; Galkin, N. G.; Pereyaslavets, L. B.; Tarasov, V. I. Quantum Mechanical Polarizable Force Field (QMPFF3): Refinement and Validation of the Dispersion Interaction for Aromatic Carbon. *J. Chem. Phys.* **2006**, *125* (24), 244107.
(4) Donchev, A. G.; Galkin, N. G.; Illarionov, A. A.; Khoruzhii, O. V.; Olevanov, M. A.; Ozrin, V. D.; Pereyaslavets, L. B.; Tarasov, V. I. Assessment of Performance of the General Purpose Polarizable Force Field QMPFF3 in Condensed Phase. *J. Comput. Chem.* **2008**, *29* (8), 1242–1249.
(5) Pearlman, D. A. A Comparison of Alternative Approaches to Free Energy Calculations. *J. Phys. Chem.* **1994**, *98* (5), 1487–1493.
(6) Woods, C. J.; Essex, J. W.; King, M. A. Enhanced Configurational Sampling in Binding Free-Energy Calculations. *J. Phys. Chem. B* **2003**, *107* (49), 13711–13718.
(7) Wang, L.; Friesner, R. A.; Berne, B. J. Replica Exchange with Solute Scaling: A More Efficient Version of Replica Exchange with Solute Tempering (REST2). *J. Phys. Chem. B* **2011**, *115* (30), 9431–9438.
(8) He, X.; Liu, S.; Lee, T.-S.; Ji, B.; Man, V. H.; York, D. M.; Wang, J. Fast, Accurate, and Reliable Protocols for Routine Calculations of Protein--Ligand Binding Affinities in Drug Design Projects Using AMBER GPU-TI with ff14SB/GAFF. *ACS Omega* **2020**, *5* (9), 4611–4619.
(9) Lu, C.; Wu, C.; Ghoreishi, D.; Chen, W.; Wang, L.; Damm, W.; Ross, G. A.; Dahlgren, M. K.; Russell, E.; Von Bargen, C. D.; Abel, R.; Friesner, R. A.; Harder, E. D. OPLS4: Improving Force Field Accuracy on Challenging Regimes of Chemical Space. *J. Chem. Theory Comput.* **2021**, *17* (7), 4291–4300.
(10) Raman, E. P.; Paul, T. J.; Hayes, R. L.; Brooks, C. L. Automated, Accurate, and Scalable Relative Protein–Ligand Binding Free-Energy Calculations Using Lambda Dynamics. *J. Chem. Theory Comput.* **2020**, *16* (12), 7895–7914.
(11) Song, L. F.; Lee, T.-S.; Zhu, C.; York, D. M.; Merz, K. M., Jr. Using AMBER18 for Relative Free Energy Calculations. *J. Chem. Inf. Model.* **2019**, *59* (7), 3128–3135.
(12) He, X.; Liu, S.; Lee, T.-S.; Ji, B.; Man, V. H.; York, D. M.; Wang, J. Fast, Accurate, and Reliable Protocols for Routine Calculations of Protein–Ligand Binding Affinities in Drug Design Projects Using AMBER GPU-TI with ff14SB/GAFF. *ACS Omega* **2020**, *5* (9), 4611–4619.
(13) Baum, B.; Mohamed, M.; Zayed, M.; Gerlach, C.; Heine, A.; Hangauer, D.; Klebe, G. More than a Simple Lipophilic Contact: A Detailed Thermodynamic Analysis of Nonbasic Residues in the s1 Pocket of Thrombin. *J. Mol. Biol.* **2009**, *390* (1), 56–69.

mutation	ΔΔG (exp)	ΔΔG (MD) [A]	ΔΔG (MD) [B]	ΔΔG (MD) [A/B]	ΔΔG (MD) [res. HREX]	ΔΔG (MD) [res. NEQ)	ΔΔG (MD) [soft. A]	ΔΔG (MD) [soft. B]	ΔΔG GAFF[a]	ΔΔG OPLS4[b]	ΔΔG CGenFF[d]
27->28	-0.50	-0.96	0.20	-0.65	-0.97	-1.63	-1.04	-0.90	-0.54	0.30	-0.04
27->30	-1.73	-1.36	-0.06	-1.01	-1.21	-1.26	-0.49	-1.25	-0.74	-0.14	-0.60
27->35	-2.69	-2.56	-1.66	-2.27	-3.09	-2.44	-2.15	-2.33	-2.66	-1.42	-1.42
27->38	-0.85	-1.72	1.89	-1.31	-0.69	-1.34	-1.18	-0.77	-2.30	-2.28	-1.67
27->43	-0.91	-2.01	0.26	-1.60	-1.76	-1.16	-1.98	-1.33	-1.84	-0.41	-0.82
27->46	-1.48	-2.61	-1.32	-2.26	-2.38	-2.25	-2.04	-2.05	-2.55	-1.23	0.88
27->52	-3.11	-3.47	-1.89	-3.10	-1.79	-2.39	-2.90	-2.42	-2.63	-2.90	-2.05
27->36	-2.06	-3.15	-1.59	-2.78	-3.38	-3.11	-	-	-2.96	-1.95	-1.31
27->44	-2.55	-3.61	1.01	-3.21	-2.89	-2.85	-1.57	-2.48	-3.3	-2.40	-2.22
27->42	-2.78	-2.59	1.46	-2.16	-2.30	-2.67	-2.54	-1.55	-1.46	-1.80	-2.07
27->45	-2.83	-2.71	0.24	-2.30	-2.51	-2.75	-1.92	-2.23	-3.21	-1.37	-2.02
27->41	-1.01	-0.49	-0.49	-0.49	-0.27	-1.12	-0.49	-0.49	-2.34	-1.50	-
27->32	-0.46	0.08	0.08	0.08	-0.38	0.15	0.08	0.08	-0.82	-0.65	-0.67
27->33	-0.76	-1.16	-1.16	-1.16	-1.22	-0.85	-1.16	-1.16	-1.49	-1.31	-0.54
27->37	-2.83	-3.09	-3.09	-3.09	-2.94	-2.75	-3.09	-3.09	-3.3	-2.44	-3.25
27->39	-0.91	-0.26	-0.26	-0.26	-0.87	0.84	-0.26	-0.26	-2.61	-1.74	-2.03
27->53	-3.84	-2.73	-2.73	-2.73	-2.40	-2.50	-2.73	-2.73	-2.89	-3.69	-4.39
r	1.00	0.80	0.44	0.83	0.74	0.75	0.79	0.86	0.60	0.70	0.72
**slope**	1.00	0.74	0.33	0.83	0.79	0.72	0.88	1.00	0.52	0.67	0.85
**MAE**	0.00	0.61	1.38	0.54	0.59	0.56	0.66	0.49	0.82	0.66	0.77

Table S1. Relative binding free energy of ligands to MCL1. Values duplicated for symmetrical ligands are shown in gray. a [8], b [9], d [10]

mutation	ΔΔG (exp, ITC)	ΔΔG (exp, Ki)	ΔΔG (MD)[A]	ΔΔG (MD)[B]	ΔΔG (MD)[A/B]	ΔΔG (MD) [res. NEQ]	ΔΔG GAFF[c]	ΔΔG OPLS4[b]
5 -> 1a	0.10	-0.61	-0.26	0.76	0.06	0.35	-1.20	-0.47
5 -> 3a	-0.74	-1.32	-1.77	0.46	-1.38	-0.97	-2.24	-1.31
5 -> 1b	-0.88	-2.45	-2.28	-0.35	-1.89	-1.75	-2.63	-1.61
5 -> 6a	-1.60	-3.10	-3.49	-0.75	-3.10	-3.14	-2.82	-3.35
5 -> 6e	-1.33	-1.84	-3.03	-1.64	-2.67	-2.60	-2.56	-1.98
5 -> 7a	-0.64	-0.82	-2.45	-1.21	-2.11	-1.76	-1.97	-1.77
5 -> 3b	-0.28	-0.46	-2.08	0.75	-1.67	-0.59	-2.07	-1.57
5 -> 6b	-1.31	-2.23	-3.13	-2.41	-2.87	-2.14	-1.91	-2.27
		***r*:**	0.92	0.74	0.92	0.96	0.77	0.88
	**ITC**	**slope:**	1.62	1.52	1.63	1.90	0.69	1.27
		**MAE:**	1.47	0.78	1.12	0.80	-1.34	0.96
		***r*:**	0.71	0.52	0.71	0.81	0.73	0.77
	**Ki**	**slope:**	0.75	0.64	0.75	0.96	0.39	0.66
		**MAE:**	0.83	1.20	0.66	0.50	-0.57	0.44

Table S2. Relative binding free energy of ligands to Thrombin. c [11], b [9]

mutation	ΔΔG (exp)	ΔΔG (MD)[A]	ΔΔG (MD)[B]	ΔΔG (MD)[A/B]	ΔΔG (MD) [res. NEQ]	ΔΔG GAFF[a]	ΔΔG OPLS4[b]	ΔΔG CGenFF[d]
1h1q->1h1r	0.51	-1.67	-0.59	-1.52	-1.77	-1.32	-0.79	-0.44
1h1q->1oi9	-1.56	-0.20	-0.20	-0.20	0.29	-1.46	-1.83	-1.06
1h1q->1oiy	-1.61	-0.27	-0.27	-0.27	0.32	-1.17	-2.04	-1.65
1h1q->20	-0.54	-2.18	-0.57	-1.95	-1.35	-1.02	-1.10	-1.01
1h1q->21	0.35	-0.08	-0.51	-0.23	-0.25	-0.78	-0.06	-1.36
1h1q->22	0.32	0.65	0.11	0.43	0.12	-0.93	-0.28	-1.17
1h1q->26	-0.25	-0.05	-0.05	-0.05	0.02	-0.92	-0.88	-1.56
r	1.00	-0.04	-0.14	-0.09	-0.50	-0.79	0.93	0.54
**slope**	1.00	-0.04	-0.45	-0.09	-0.53	-0.04	0.77	0.19
**MAE**	0.00	1.07	0.73	1.00	1.13	0.85	0.60	0.92

Table S3. Relative binding free energy of ligands to CDK2. Values duplicated for symmetrical ligands are shown in gray. a [8], b [9]

mutation	ΔΔG (exp)	PIMD	ΔG (MD) [A] protein	ΔG (MD) [B] water	ΔΔG (MD)
1h1q->1oi9	-1.56	0	-3.62	-3.42	-0.20
1h1q->1oi9	-1.56	4	-3.23	-2.97	-0.26
1h1q->1oiy	-1.61	0	-13.21	-12.94	-0.27
1h1q->1oiy	-1.61	4	-12.65	-12.39	-0.25

Table S4. Relative binding free energy of two ligands to CDK2 determined with PIMD=4, taking nuclear quantum effect into account, and without it (same as in Table. S3).

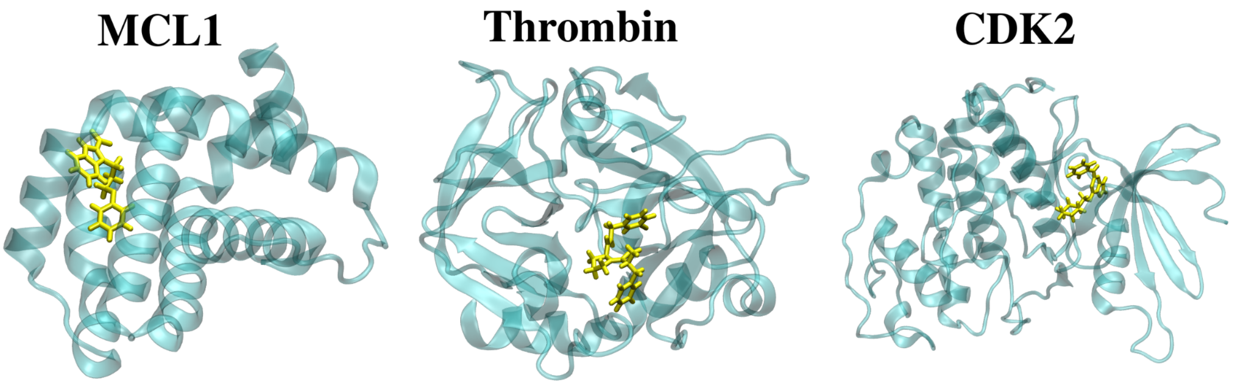

Figure S1. Proteins with reference ligands: MCL1 with ligand 27, Thrombin with ligand 5, and CDK2 with ligand 1h1q.

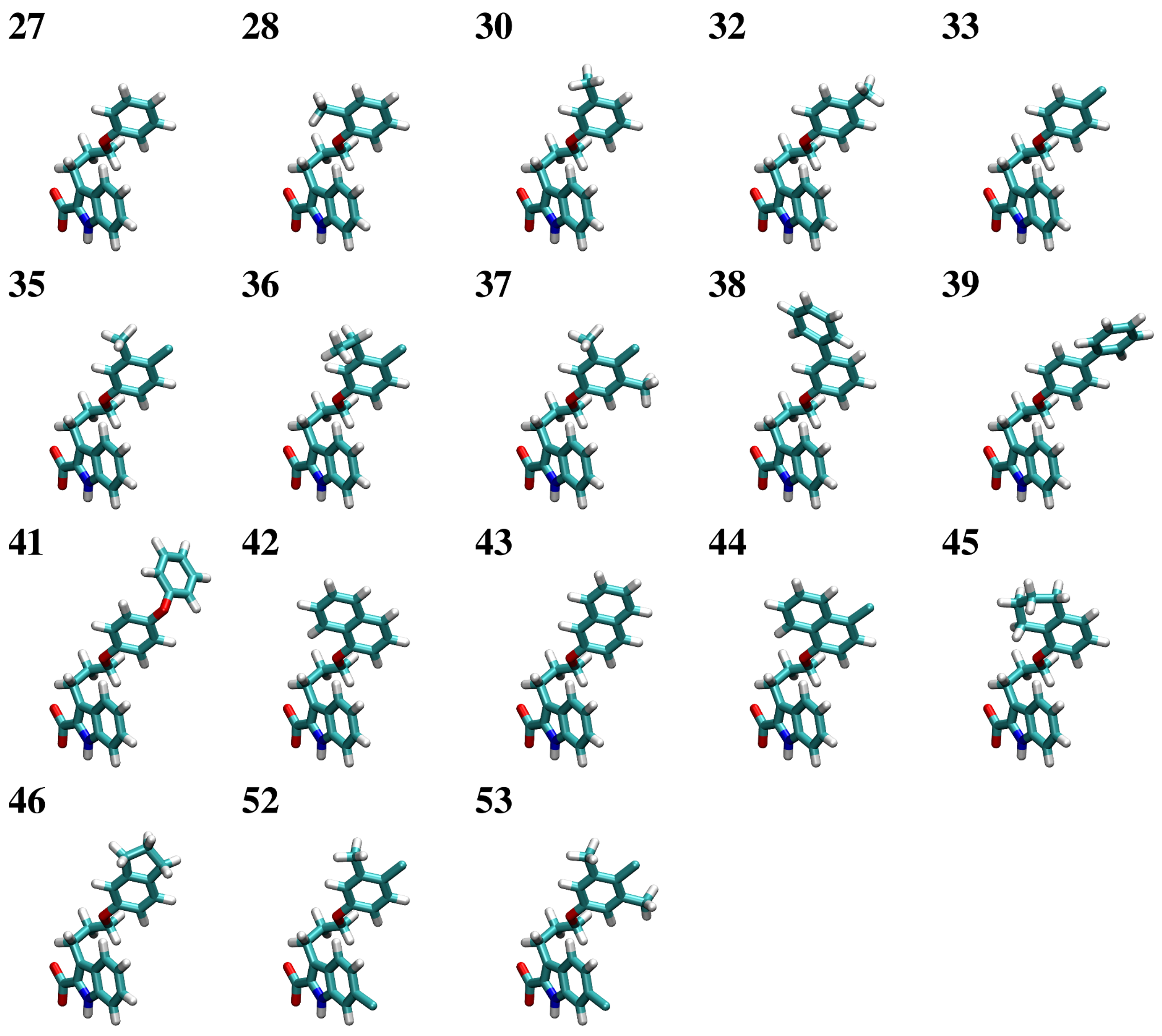

Figure S2. Ligands for MCL1.

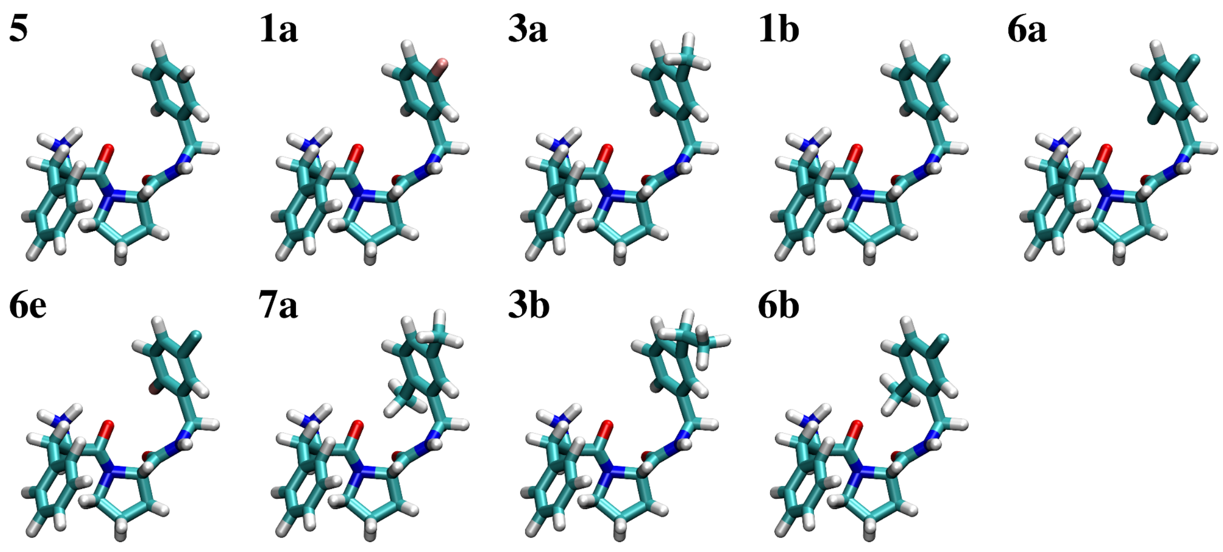

Figure S3. Ligands for Thrombin.

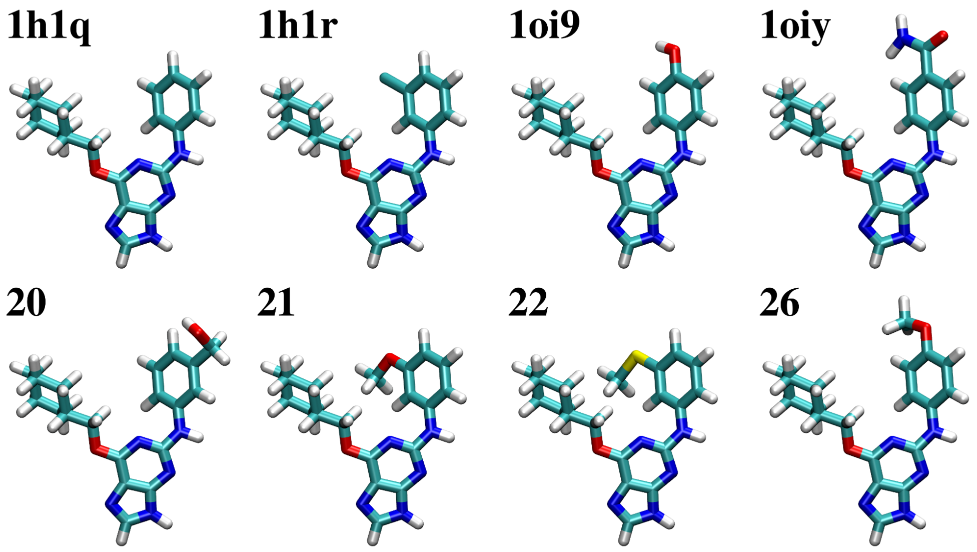

Figure S4. Ligands for CDK2.

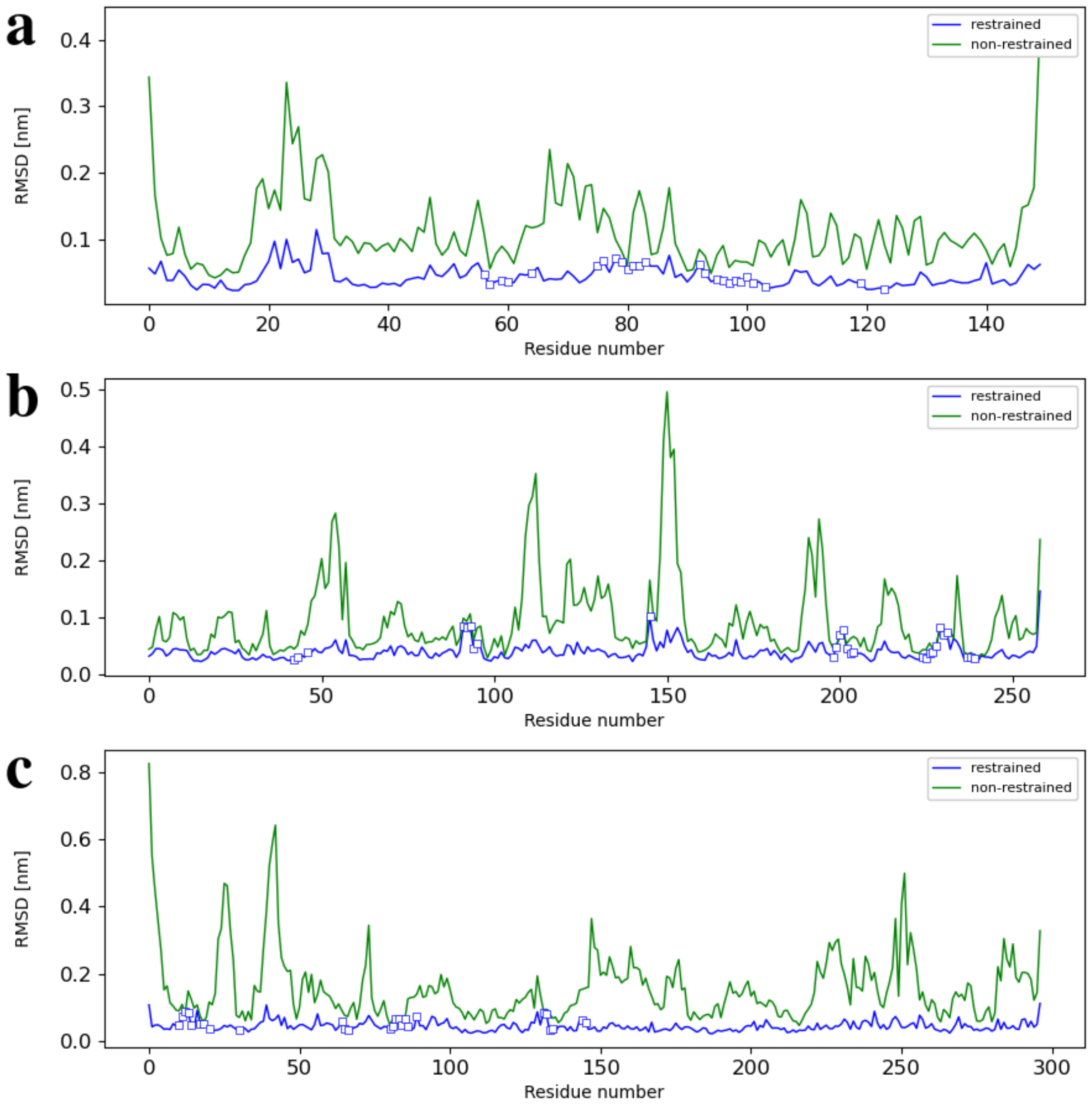

Figure S5. Root mean squared displacement (RMSD) of C_α atoms from the X-ray structures for **a)** MCL1 with ligand 27, **b)** Thrombin with ligand 6a, and **c)** CDK2 with ligand 1h1q. The RMSD was calculated from restrained and non-restrained simulations. In the restrained simulations, the C_α atoms of the binding pocket that were not restrained are shown by empty squares.

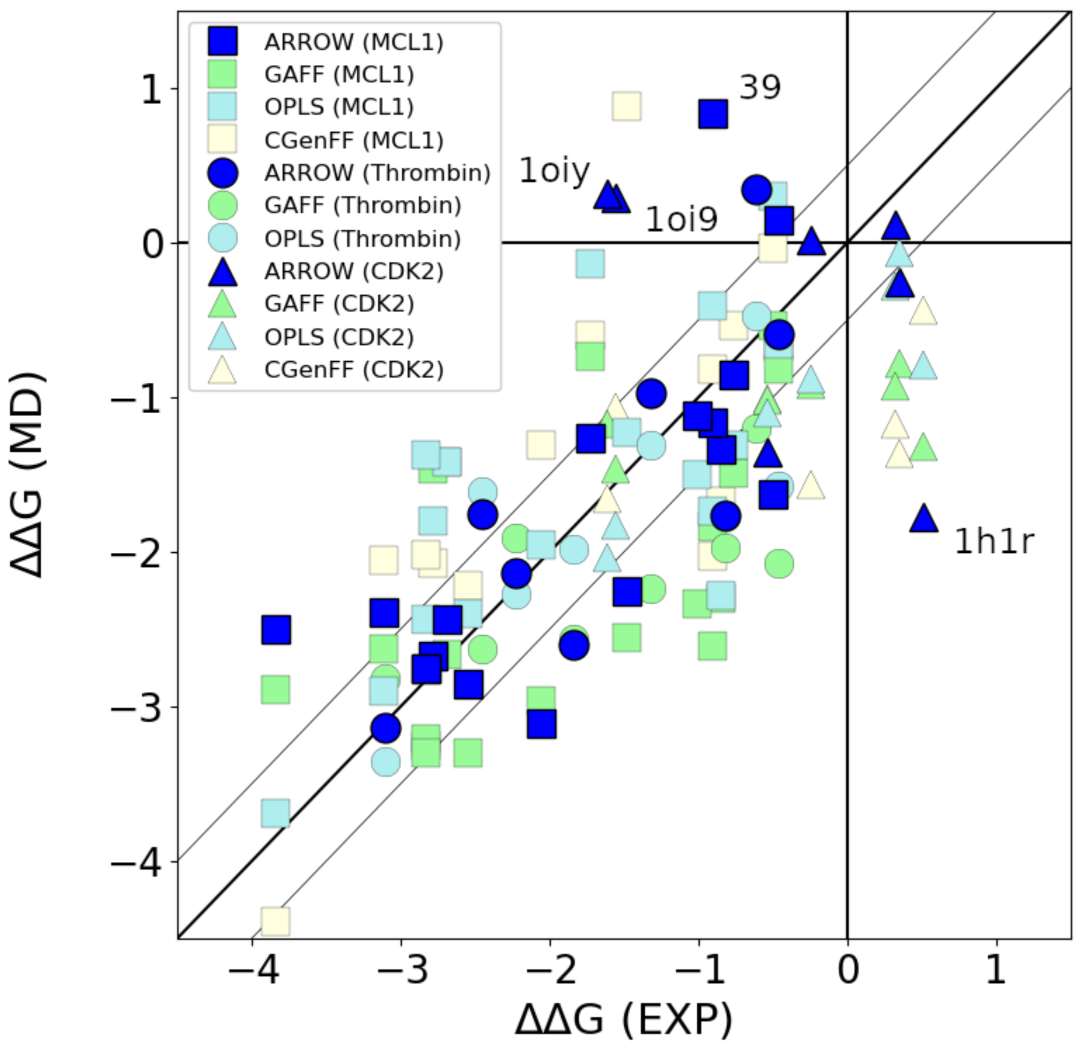

Figure S6. A parity plot comparing the relative binding free energies ΔΔG for ligand mutations in MCL1, Thrombin, and CDK2 as predicted by ARROW FF and experiment. Also shown for comparison are the predictions of GAFF [11,12], OPLS [9], and CGenFF [10] force fields. Results with ARROW were calculated with HREX and conformation reservoir generated *via* potential softening and NEQ MD. Selected ARROW ΔΔG values with the largest deviation from the experiment (MAE > 1.5 kcal/mol) are marked with labels - ligands 1h1r, 1oi9, and 1oiy in CDK2, and ligand 39 in MCL1. The thin gray lines are +/- 0.5 kcal/mol from the diagonal.

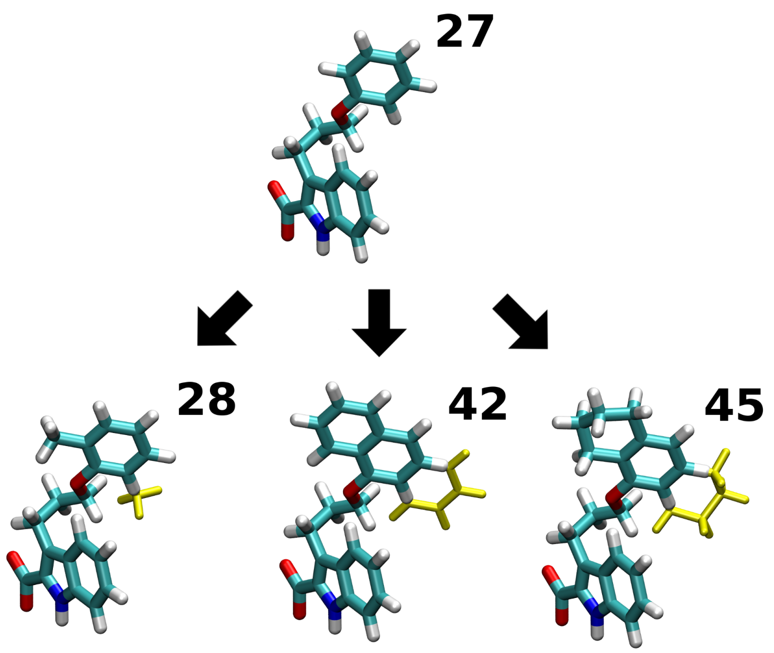

Figure S7. Examples of asymmetrical ligand mutations in MCL1. Alternative initial orientations of the mutated sites are shown in yellow.

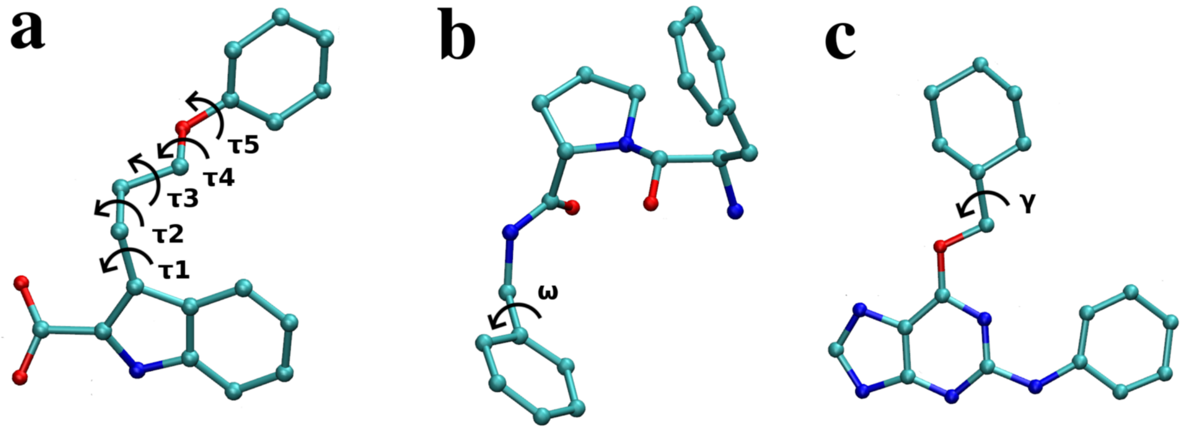

Figure S8. Ligand **a)** 27 of MCL1, **b)** 5 of Thrombin, and **c)** 1h1q of CDK2. Hydrogen atoms are not shown. Rotatable torsions softened to enhance sampling are shown with labels - $\tau 1, 2, 3, 4, 5$ for ligand 27, ω for ligand 5, and γ for 1h1q.

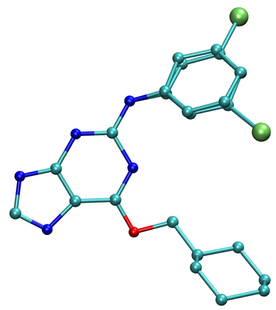

Figure S9. Alternative conformations of ligand 1h1r (chlorobenzene site) in CDK2 from X-ray structure (PDB: 1h1r, chain A).

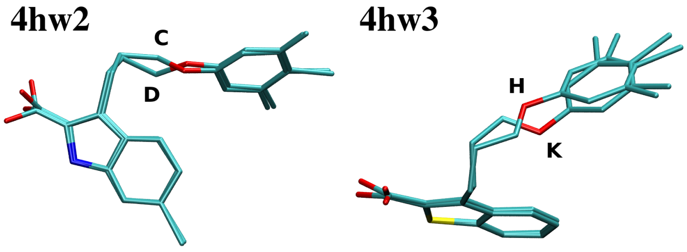

Figure S10. Selected conformations of ligands 53 and 60 in MCL1 from X-ray structures (PDB: 4hw2 and 4hw3, respectively) superimposed on each other. The conformations are labeled by their chain ID’s.

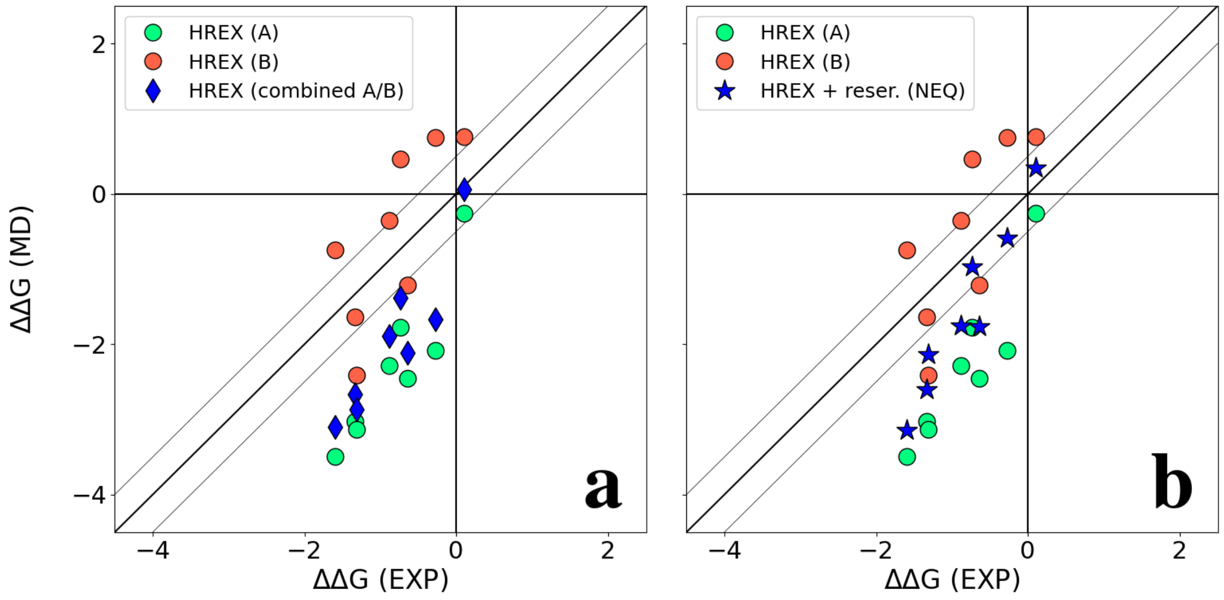

Figure S11. Comparison of ΔΔG as determined with ARROW force field and ITC experiment [13] for Thrombin. Green and red markers correspond to values obtained with HREX and a target ligand starting at A and B sides, respectively. Blue markers correspond to **a)** combined A/B sides, **b)** HREX with reservoir (from NEQ). Thin gray lines are +/- 0.5 kcal/mol from the diagonal.

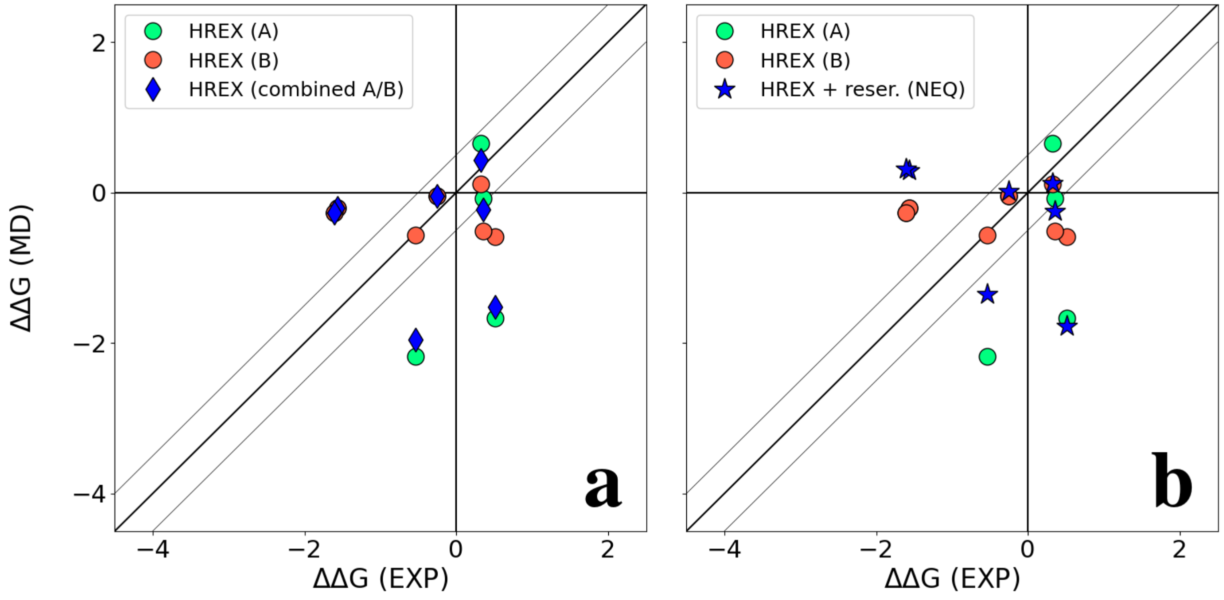

Figure S12. Comparison of ΔΔG as determined with ARROW force field and experiment for CDK2. Green and red markers correspond to values obtained with HREX and a target ligand starting at A and B sides, respectively. Blue markers correspond to **a)** combined A/B sides, **b)** HREX with reservoir (from NEQ). Thin gray lines are +/- 0.5 kcal/mol from the diagonal.

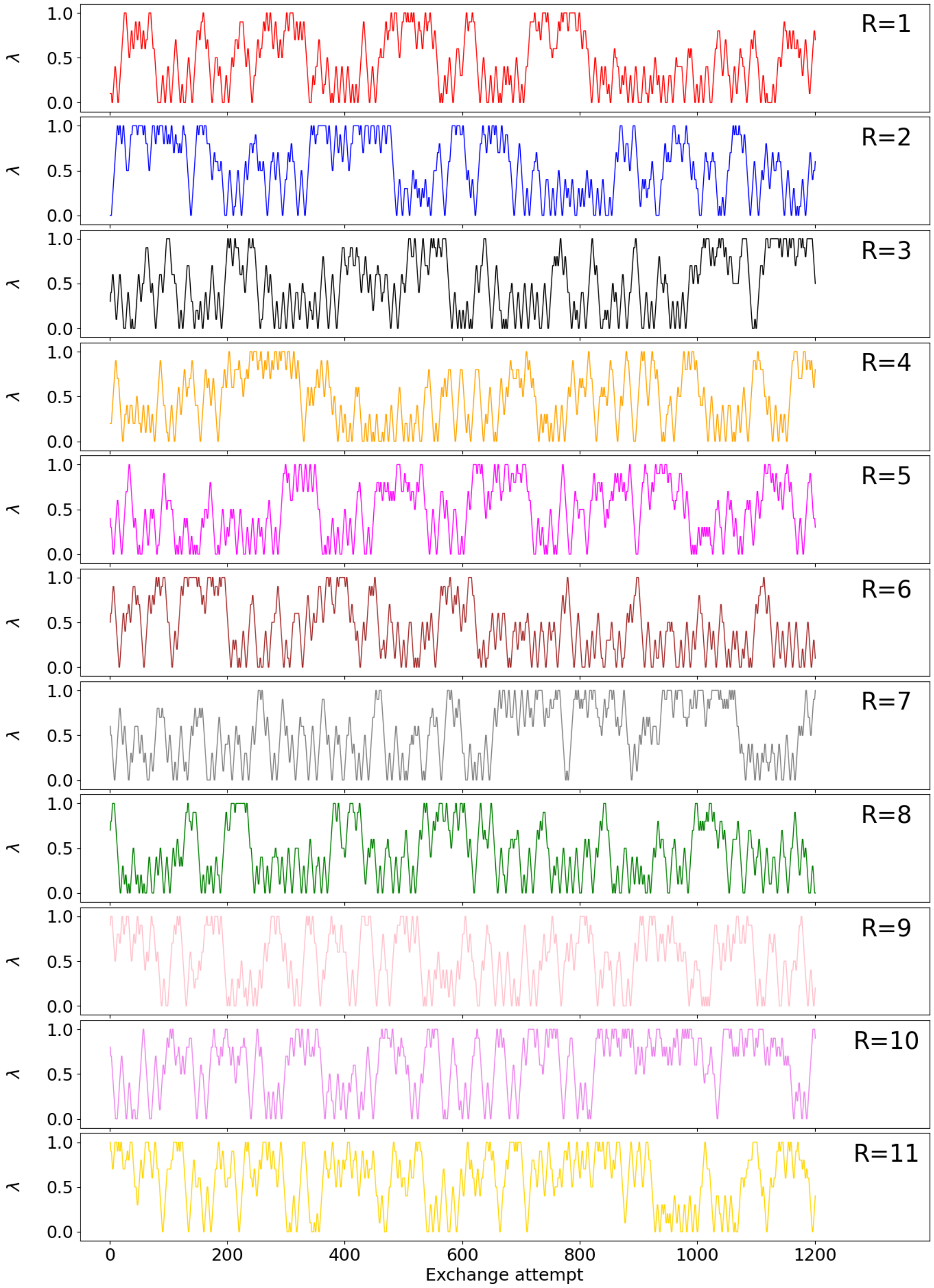

Figure S13. Dynamics of 11 system replicas in the space of Hamiltonians spanning from λ=0.0 (ligand 27) to λ=1.0 (ligand 30) in MCL1 protein during HREX simulation. A conformation reservoir (from NEQ) was attached to λ=0.0 replica. The neighboring replicas are exchanging with the following rates: 0.0↔0.1: 0.85, 0.1↔0.2: 0.89, 0.2↔0.3: 0.90, 0.3↔0.4: 0.77, 0.4↔0.5: 0.67, 0.5↔0.6: 0.66, 0.6↔0.7: 0.65, 0.7↔0.8: 0.69, 0.8↔0.9: 0.64, 0.9↔1.0: 0.59.

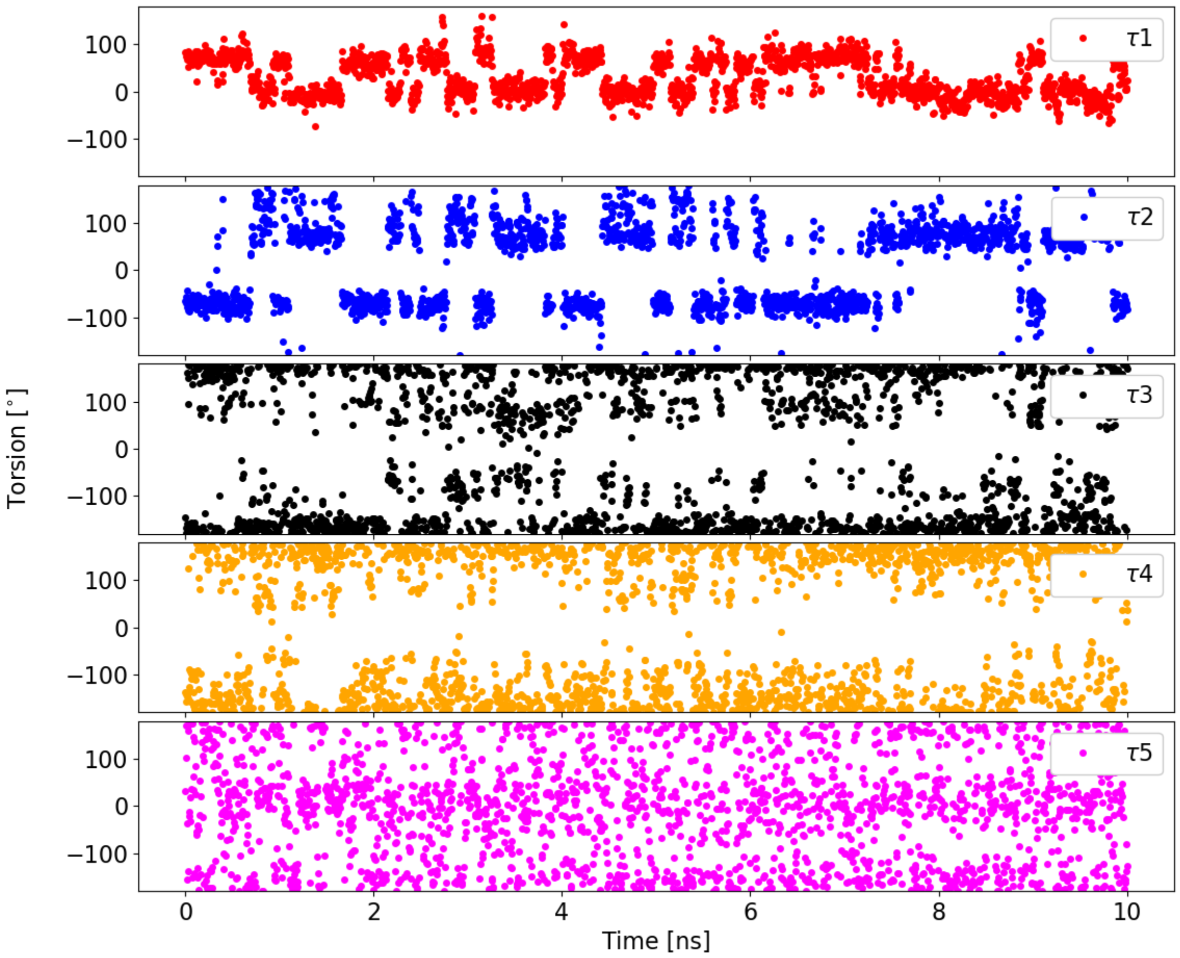

Figure S14. Dynamics of rotable torsions of ligand 27 in MCL1 protein during 10 ns MD with softened potential.

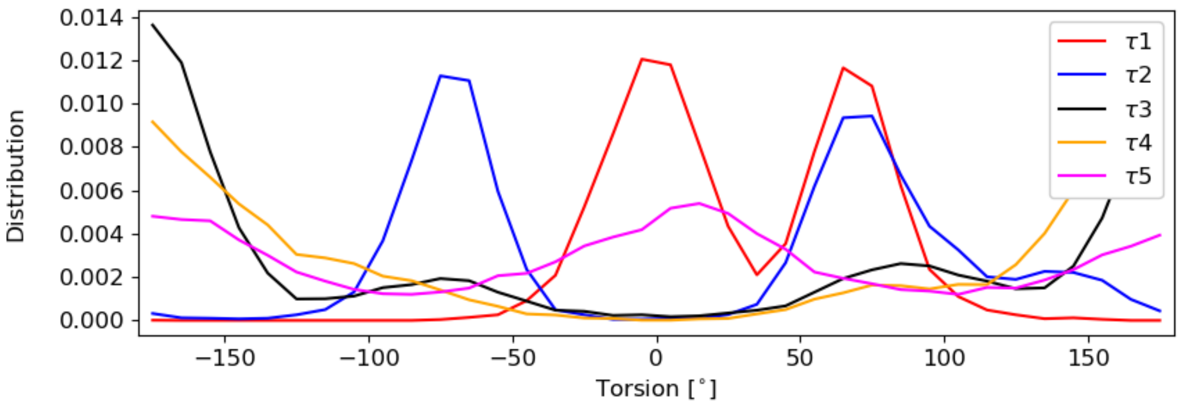

Figure S15. Distribution of rotable torsions of ligand 27 in MCL1 protein during 10 ns MD with softened potential.
